# Monocyte/Macrophage-Specific Loss of ARNTL Suppresses Chronic Kidney Disease-Associated Cardiac Impairment

**DOI:** 10.3390/ijms252313009

**Published:** 2024-12-03

**Authors:** Yuya Yoshida, Naoki Nishikawa, Kohei Fukuoka, Akito Tsuruta, Kaita Otsuki, Taiki Fukuda, Yuma Terada, Tomohito Tanihara, Taisei Kumamoto, Ryotaro Tsukamoto, Takumi Nishi, Kosuke Oyama, Kengo Hamamura, Kouta Mayanagi, Satoru Koyanagi, Shigehiro Ohdo, Naoya Matsunaga

**Affiliations:** 1 Department of Clinical Pharmacokinetics, Faculty of Pharmaceutical Sciences, Kyushu University, 3-1-1 Maidashi, Higashi-ku, Fukuoka 812-8582, Japanotsuki.kaita.518@s.kyushu-u.ac.jp (K.O.); fukuda.taiki.747@s.kyushu-u.ac.jp (T.F.); tsukamoto.ryotaro.843@s.kyushu-u.ac.jp (R.T.); nishi.takumi.735@s.kyushu-u.ac.jp (T.N.); hamamura@phar.kyushu-u.ac.jp (K.H.); ohdo@phar.kyushu-u.ac.jp (S.O.); 2 Department of Pharmaceutics, Faculty of Pharmaceutical Sciences, Kyushu University, 3-1-1 Maidashi, Higashi-ku, Fukuoka 812-8582, Japan; tsuruta@phar.kyushu-u.ac.jp (A.T.); koyanagi@phar.kyushu-u.ac.jp (S.K.); 3 Department of Biological Science and Technology, Faculty of Advanced Engineering, Tokyo University of Science, 1-3 Kagurazaka, Shinjuku-ku, Tokyo 162-8601, Japan; k.oyama@rs.tus.ac.jp; 4 Department of Drug Discovery Structural Biology, Faculty of Pharmaceutical Sciences, Kyushu University, 3-1-1 Maidashi, Higashi-ku, Fukuoka 812-8582, Japan

**Keywords:** ARNTL, chronic kidney disease, cardiac pathology GPR68, circadian clock mechanism

## Abstract

Defects in Aryl hydrocarbon receptor nuclear translocator-like 1 (ARNTL), a central component of the circadian clock mechanism, may promote or inhibit the induction of inflammation by monocytes/macrophages, with varying effects on different diseases. However, ARNTL’s role in monocytes/macrophages under chronic kidney disease (CKD), which presents with systemic inflammation, is unclear. Here, we report that the expression of *Arntl* in monocytes promoted CKD-induced cardiac damage. The expression of G-protein-coupled receptor 68 (GPR68), which exacerbates CKD-induced cardiac disease, was regulated by ARNTL. Under CKD conditions, GPR68 expression was elevated via ARNTL, particularly in the presence of PU.1, a transcription factor specific to monocytes and macrophages. In CKD mouse models lacking monocyte-specific ARNTL, GPR68 expression in monocytes was reduced, leading to decreased cardiac damage and fibrosis despite no improvement in renal excretory capacity or renal fibrosis and increased angiotensin II production. The loss of ARNTL did not affect the expression of marker molecules, indicating the origin or differentiation of cardiac macrophages, but affected GPR68 expression only in cardiac macrophages derived from mature monocytes, highlighting the significance of the interplay between GPR68 and ARNTL in monocytes/macrophages and its influence on cardiac pathology. Understanding this complex relationship between circadian clock mechanisms and disease could help uncover novel therapeutic strategies.

## 1. Introduction

Chronic kidney disease (CKD) leads to various complications, such as cardiovascular and cerebrovascular diseases, and poses an immense global public health concern [1,2]. The morbidity and mortality rates associated with CKD are alarmingly high, with cardiovascular disease being the primary cause of death [3,4]. As renal function declines, the prevalence of cardiovascular disease increases, leading to a 10- to 100-fold rise in cardiovascular mortality risk among patients with CKD compared to that in patients without CKD [5,6]. Therefore, it is crucial to prevent cardiovascular disease in patients with CKD and develop new therapeutic approaches to enhance survival rates in patients with the disease. Currently, exhaustive and comprehensive research is underway to identify effective therapeutic approaches for managing cardiovascular disease, which is intricately intertwined with the multifaceted pathogenesis of CKD. Cardiovascular disease in patients with CKD emanates from hemodynamic overload, as well as endocrine and metabolic irregularities [7,8,9]. Additionally, inflammatory reactions associated with humoral and cellular immune responses play a pivotal role in driving the development of the disease [10,11,12].

Many mammals, including humans, exhibit physiological functions that follow a 24 h cycle known as the circadian rhythm. This rhythm is regulated by a feedback loop mechanism involving transcription and translation, known as the circadian clock mechanism, which relies on a group of transcription factors called clock genes [13]. The circadian clock mechanism exists in individual cells from the central to the peripheral nervous system, rhythmically regulating the functions of various organs. Two central clock genes, *CLOCK* and Aryl hydrocarbon receptor nuclear translocator-like 1 (*ARNTL*, also known as *BMAL1*), play a crucial role in this mechanism. CLOCK and ARNTL form heterodimers and promote transcription through a specific sequence called the E-box on *PER* and *CRY*. The resulting PER and CRY proteins are translocated into the nucleus when their intracellular concentration reaches a certain level, and their transcriptional activity is cyclically repressed by the CLOCK/ARNTL complex [14,15]. This mechanism also acts on target genes coding for enzymes, receptors, and other factors crucial for biological functions, triggering 24 h cycles to maintain homeostasis [13,16]. Therefore, chronic alterations or the dysfunction of clock genes have been implicated in the onset and pathogenesis of various diseases. Numerous epidemiological and animal studies have demonstrated a close association between circadian clock mechanisms and cardiovascular function, as well as the development of cardiovascular events [17,18,19,20]. These studies suggest that abnormalities in circadian clock mechanisms are also involved in cardiovascular homeostasis during CKD.

In response to the C-C motif chemokine ligand 2 produced by damaged tissues, monocytes migrate to the sites of inflammation and differentiate into macrophages, playing a vital role in tissue inflammation and tissue repair [21,22]. A correlation between monocyte infiltration and the pathophysiology of CKD has also been previously reported [23,24]. In our previous study, we demonstrated that G-protein-coupled receptor 68 (GPR68), induced in monocytes during CKD, aggravates cardiac inflammation [10]. In addition, elevated GPR68 expression has been implicated in retinol-induced alterations in the circadian clock mechanism [10,25]. These findings highlight the significance of circadian clock mechanisms for monocytes and macrophages in cardiac pathogenesis during CKD. However, the cell specificity of CKD-induced GPR68 expression is poorly understood. This emphasizes the need for further in-depth analyses of these relationships.

*ARNTL* is distinct from other clock genes, such as *CLOCK*, *PER*, and *CRY*, as no other molecules are capable of replacing its subtype or function. Consequently, the loss of *ARNTL* disrupts the circadian clock mechanism, resulting in the elimination of circadian oscillations in gene expression [26]. The impact of ARNTL defects in monocytes and macrophages varies widely from one disease to another, affecting the progression of conditions such as melanoma [27], sepsis [28], atherosclerotic lesions [29], and skeletal muscle repair [30]. Depending on the disease, these defects can either promote or inhibit inflammation and immune cell mobilization. This highlights the influence of ARNTL on monocyte/macrophage function and demonstrates that its intracellular function is greatly influenced by the specific environment created by each disease. Investigations in mice with systemic mutations of CLOCK, which forms a heterodimer with ARNTL, have shown that systemic CLOCK affects the outcome of CKD-induced fibrosis in both the heart and kidneys [10,31]. This indicates that the loss of monocyte/macrophage-specific ARNTL in CKD conditions may affect both cardiac and renal dysfunction and that CKD-induced GPR68 expression may be associated with this dysfunction. Accordingly, analyses focusing on these proteins are expected to contribute significantly to our understanding of the diversity of monocyte/macrophage ARNTL involvement in the heart, kidneys, and other organs.

In the present study, we aimed to investigate the cause of monocyte/macrophage-specific GPR68 upregulation in CKD and the relationship between CKD-induced cardiac and renal impairment and ARNTL in monocytes and macrophages. Initially, we analyzed the contribution of E-box and ARNTL to the monocyte/macrophage specificity of GPR68 expression using mouse monocyte/macrophage-like cells (RAW264.7) and primary monocytes. Additionally, we utilized 5/6 nephrectomized (5/6Nx) mice as a model of CKD, as these mice were shown to exhibit correlations with various factors observed in patients with CKD in our previous studies [10,31,32,33]. We generated 5/6 nephrectomized monocyte-specific *Arntl*-knockout (KO) mice by crossing *Lyz2*-CRE mice with *Arntl*-loxp mice and analyzed the expression of marker molecules, including GPR68 in tissue macrophages, and cardiac and renal pathology. These analyses revealed that GPR68 expression in monocytes/macrophages is dependent on the expression of not only ARNTL but also PU.1, a transcription factor that is specifically expressed in monocytes/macrophages. Furthermore, the loss of monocyte/macrophage-specific ARNTL suppressed the upregulation of GPR68 expression in monocytes by 5/6Nx, and this was accompanied by the suppression of cardiac inflammation and fibrosis. In contrast, the loss of monocyte/macrophage-specific ARNTL did not suppress renal impairment. The number of monocytes and macrophages in both organs and the expression of macrophage marker molecules were unaffected by the loss of ARNTL, but the expression of GPR68 in macrophages differed between the kidneys and heart. These new findings provide new evidence of the relationship between CKD, monocytes/macrophages, ARNTL, and renal/cardiac impairment, which has not been fully elucidated in previous studies about CKD [10,25,31,32,33,34].

## 2. Results

### 2.1. Mechanism Underlying GPR68 Upregulation and Cell Specificity in RAW264.7 and Mouse Primary Monocytes

Multiple E-boxes upstream of the GPR68-encoding gene in both mice and humans (Figure S1A) suggest that GPR68 expression may exhibit circadian oscillations regulated by the circadian clock mechanism. To investigate this phenomenon, we examined the circadian oscillations of GPR68 expression in the mouse monocyte/macrophage-like cell line RAW264.7 and the human monocyte cell line THP-1. Upon treatment with 100 nmol/L dexamethasone (DEX) [35], the mRNA expression of PER1, ARNTL, and GPR68 showed significant circadian oscillations (*p* < 0.05; Figure S1B,C). Furthermore, Gpr68 expression in Arntl-KO RAW264.7 cells showed no significant difference between 36 and 48 h after DEX treatment (*p* < 0.01; Figure S2).

**Figure 1:**
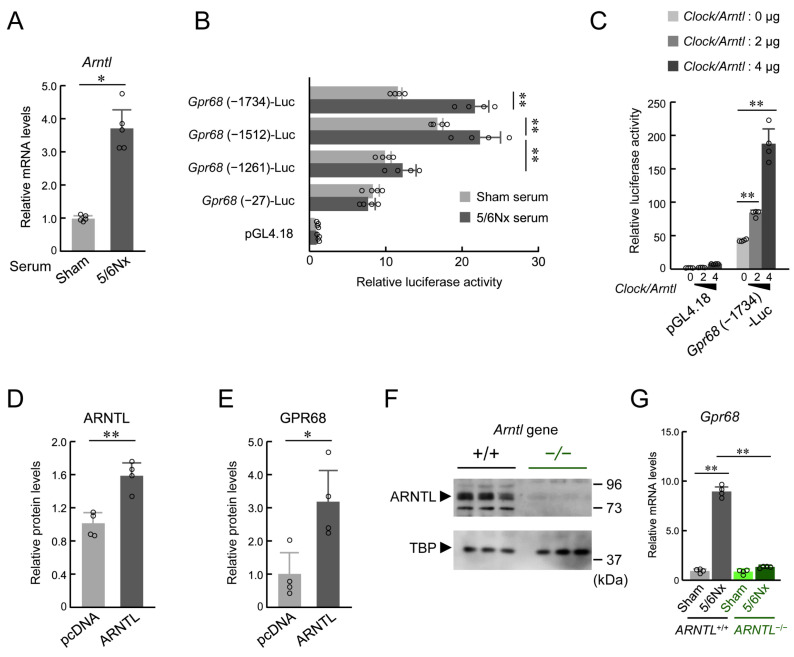
Effect of ARNTL on induction of GPR68 expression in RAW264.7 and mouse primary monocytes by 5/6Nx-derived serum. (**A**) mRNA of *Arntl* in RAW264.7 incubated with 10% serum from Sham and 5/6Nx mice for 24 h. (**B**) Transcriptional regulation of *Gpr68* using serum prepared from Sham or 5/6Nx mice. Number of nucleotide residues indicates distance from transcription start site (+1). RAW264.7 cells were transfected with *Gpr68* (-1734)-Luc, *Gpr68* (-1512)-Luc, *Gpr68* (-1261)-Luc, *Gpr68* (-27)-Luc, or pGL4.18. Values are expressed as mean ± S.D. (*n* = 4). (**C**) Influence of CLOCK/ARNTL on transcriptional activity of mouse *GPR68*. RAW264.7 cells were transfected with *Gpr68* (-1734)-Luc in presence or absence of CLOCK and ARNTL-expressing vectors. Relative luciferase activity of pGL4.18-transfected cells in absence of CLOCK/ARNTL was set at 1.0. (**D**) High-ARNTL-expressing RAW264.7 was created by introducing an ARNTL expression plasmid. ARNTL expression levels were measured using Western blotting. (**E**) Protein levels of GPR68 in RAW264.7-transfected pcDNA3.1 or ARNTL-expressing vectors. (**F**) Loss of *Arntl* caused by CRE-LOXP system resulted in loss of ARNTL protein in monocytes. Monocytes isolated from monocytic *ARNTL* +/+ mice or monocytic *ARNTL* −/− mice. (**G**) Expression of *Gpr68* mRNA in primary cultured monocytes, which were isolated from monocytic *ARNTL* +/+ mice or monocytic *ARNTL* −/− mice. mRNA levels of *Gpr68* were assessed after treatment with serum from Sham or 5/6Nx WT mice for 24 h. Values are expressed as mean ± S.D. (*n* = 4–6). *, *p* < 0.05, **, *p* < 0.01 indicates significant differences between two groups (two-way ANOVA with Tukey–Kramer post hoc tests or Student’s *t*-test).

GPR68 expression in monocytes is induced by the retinol/RBP4 complex in 5/6Nx serum [10]. To further analyze the relationship between ARNTL and GPR68 expression under 5/6Nx conditions, we first investigated whether RAW264.7 cells showed the same upregulation of GPR68 by 5/6Nx serum via STRA6. When STRA6 was not downregulated, GPR68 expression in RAW264.7 cells was upregulated when cultured in medium supplemented with 10% serum from wild-type 5/6Nx mice (Figure S3A,B). In contrast, when STRA6 was downregulated using siRNA, GPR68 expression was not upregulated (Figure S3B). Similar results were observed for Tnfα, a downstream signaling cytokine of GPR68 (Figure S3C), and ARNTL, a transcription factor thought to induce GPR68 transcription (Figure S3D). Because these results indicate that GPR68 expression was induced via retinol in 5/6Nx serum via the same mechanism as that in monocytes, we continued the validation using RAW264.7 cells. When RAW264.7 cells expressing a luciferase reporter vector containing a 1500 bp upstream region of the transcription start site of mouse Arntl were exposed to serum prepared from wild-type Sham and 5/6Nx mice, luciferase activity was increased by Arntl reporter vectors (Figures S4 and S5). Treatment with 5/6Nx serum also led to the upregulation of Arntl mRNA (Figure 1A). These results suggest that 5/6Nx serum induced Arntl transcription in RAW264.7 cells. Subsequently, we examined the relationship between the upregulation of Gpr68 and ARNTL under 5/6Nx serum. When RAW264.7 cells expressing the luciferase reporter vector containing 1734, 1512, 1261, or 27 bp upstream regions of the transcription start site of mouse Gpr68 were exposed to 5/6Nx serum, an increase in luciferase activity from Gpr68 reporter vectors containing 1734 and 1512 bp upstream regions was observed 24 h after exposure, but this increase was not observed for reporter vectors with sequences shorter than 1261 bp upstream, eliminating the E-box (Figure S4 and Figure 1B). Therefore, we investigated the relationship between ARNTL expression and Gpr68 transcription. Luciferase activity was found to increase in a plasmid-dose-dependent manner 24 h after the transfection of the mouse CLOCK/ARNTL expression plasmid into RAW264.7 cells expressing Gpr68 reporter vectors (Figure S6 and Figure 1C). The expression of the GPR68 protein was higher in cells stably expressing ARNTL than in those stably expressing pcDNA3.1 (Figure 1D,E). These results suggest that ARNTL expression facilitated GPR68 expression via the E-box upstream of Gpr68. Furthermore, to directly assess whether the ARNTL-dependent upregulation of GPR68 expression observed in RAW264.7 cells is observed in mouse monocytes, we used primary cultured monocytes collected from the circulating blood of mice expressing or lacking Arntl (Figure S7). ARNTL expression in monocytes collected from the blood of monocyte-specific ARNTL-deficient (ARNTL −/−) ICR mice was determined using Western blotting, and no bands derived from ARNTL were observed in monocytes from monocyte ARNTL-expressing (ARNTL +/+) ICR mice (Figure 1F). Exposure to wild-type 5/6Nx-derived serum did not induce Gpr68 expression in mouse primary monocytes lacking Arntl (Figure 1G). These results suggested that ARNTL is essential for the induction of Gpr68 expression in 5/6Nx mice.

**Figure 2:**
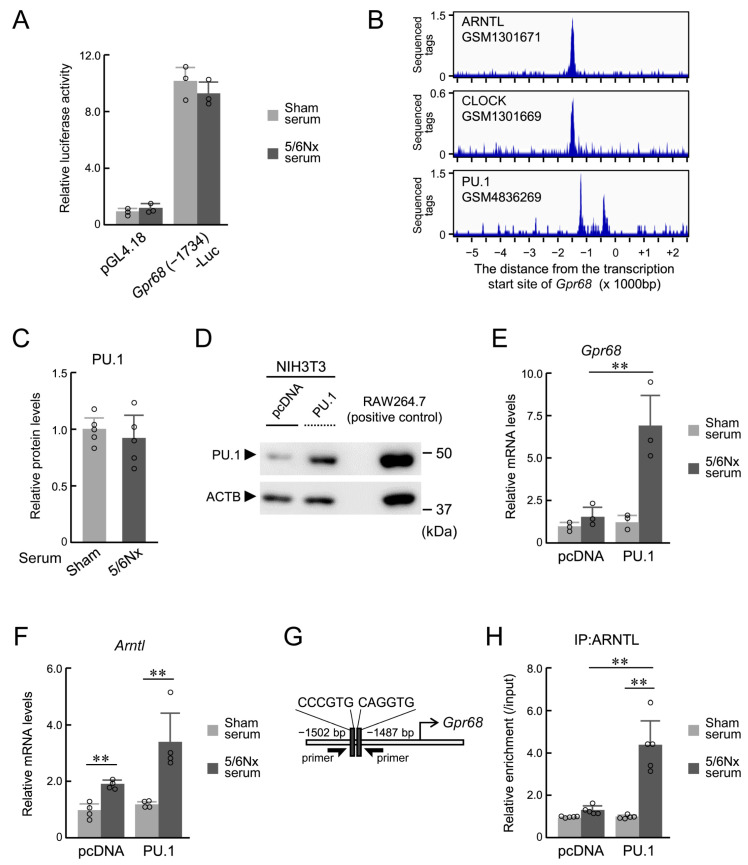
The effect of monocyte/macrophage-specific transcription factor PU.1 on the induction of GPR68 expression. (**A**) The 5/6Nx-derived serum did not increase the transcriptional activity upstream of *Gpr68* in NIH3T3. NIH3T3 was transfected with *Gpr68* (-1734)-Luc or pGL4.18 and incubated with 10% serum from Sham and 5/6Nx mice for 24 h. (**B**) Transcription factors binding upstream of *Gpr68* analyzed by previous transcriptome analyses. The blue waveform shows the sequenced tags in ChIP sequence analysis for each transcription factor. The numbers on the horizontal axis indicate the distance from the transcription start site (kbp). (**C**) The PU.1 protein in RAW264.7 incubated with 10% serum from Sham and 5/6Nx mice for 24 h. (**D**) High-PU.1-expressing NIH3T3 was created by introducing a PU.1 expression plasmid. PU.1 expression levels were measured using Western blotting. (**E**,**F**) The mRNA levels of *Gpr68* (**E**) and *Arntl* (**F**) in NIH3T3-transfected pcDNA3.1 or PU.1-expressing vectors were measured after incubation with 10% serum from Sham and 5/6Nx mice for 24 h. (**G**) A schematic of mouse *Gpr68*. The numbers indicate the distance from the transcription start site (+1). Black rectangles, E-box. The arrow symbols indicate the location on the gene where the primer sets localize for the analysis of ChIP. (**H**) The binding of endogenous ARNTL to the *Gpr68* upstream region in NIH3T3-transfected pcDNA3.1 or PU.1-expressing vectors. Values are expressed as the mean ± S.D. (*n* = 3–5). **, *p* < 0.01 indicates significant differences between the two groups (two-way ANOVA with Tukey–Kramer post hoc tests or Student’s *t*-test).

In contrast with RAW264.7 cells and mouse monocytes, when the mouse fibroblast cell line NIH3T3 was exposed to wild-type 5/6Nx-derived serum, no increase in the transcriptional activity of Gpr68 was observed (Figure 2A). In macrophages, ARNTL co-localizes with PU.1 [36], a transcription factor specifically expressed in macrophages and monocyte-derived cells (Figure S8). The co-localization of these transcription factors was investigated using three transcriptome datasets (GSM1301671, GSM1301669, and GSM4836269). In addition to ARNTL and CLOCK binding, PU.1 binding was observed in the region spanning from a position close to the transcription start point of Gpr68 and 1500 bp upstream (Figure 2B). To determine whether PU.1 expression is involved in the increased GPR68 expression observed in RAW264.7 cells, the amount of PU.1 protein in the nucleus was measured in RAW264.7 cells exposed to 5/6Nx serum. However, nuclear PU.1 expression was not altered by 5/6Nx serum (Figure 2C). To investigate the association between Gpr68 and PU.1, PU.1- or pcDNA3.1-expressing NIH3T3 cells were generated (Figure 2D). In contrast to that in pcDNA3.1-expressing cells, Gpr68 expression in PU.1-expressing NIH3T3 cells was significantly increased by exposure to wild-type 5/6Nx-derived serum (*p* < 0.05; Figure 2E). In contrast, Arntl mRNA expression was upregulated by 5/6Nx serum with or without PU.1 expression (Figure 2F). Nevertheless, the binding of ARNTL upstream of Gpr68, as measured using chromatin immunoprecipitation (ChIP) experiments (Figure 2G), was not increased in pcDNA3.1-expressing cells and exhibited a significant 5/6Nx serum-dependent increase in PU.1-expressing cells only (*p* < 0.05; Figure 2H). Because PU.1 expression is essential for the differentiation and function of bone marrow-derived cells, including monocytes/macrophages, the loss of the PU.1 gene results in the death or considerable dysfunction of these cells [37,38]. Therefore, we performed additional investigations using RAW264.7 cells in which PU.1 expression was reduced by suppressing the expression of C/EBPα, which upregulates PU.1 expression [38,39], using miRNA against Cebpa mRNA (miCebpa) (Figure S9A). In contrast to the results seen in control cells, miCebpa transfection suppressed 5/6Nx mouse serum-dependent Gpr68 upregulation (Figure S9B). These results indicate that PU.1 was involved in the induction of Gpr68 expression by ARNTL under 5/6Nx conditions, representing a monocyte- and macrophage-specific phenomenon.

### 2.2. Effect of Loss of Monocyte-Specific ARNTL on GPR68 Expression in 5/6Nx Monocytes

**Figure 3:**
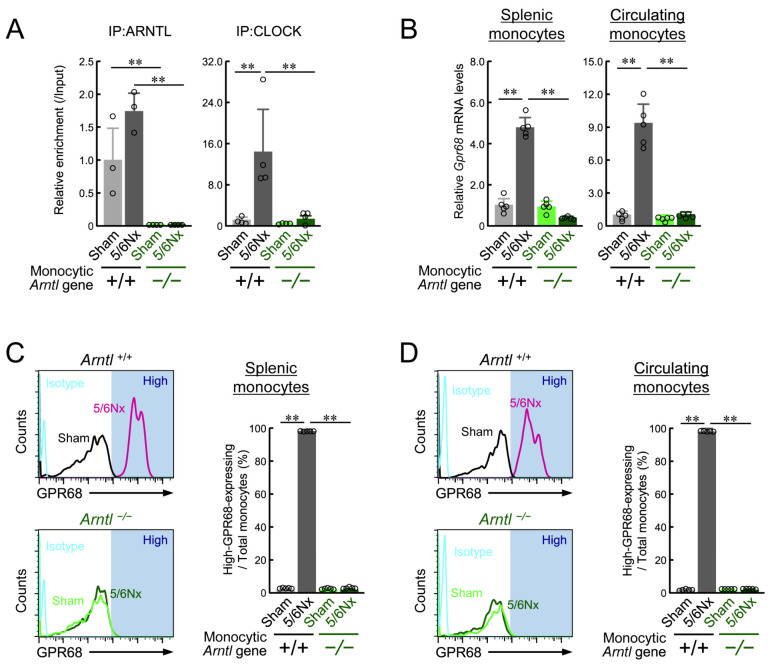
The effect of the loss of monocyte-specific ARNTL on the 5/6Nx-induced induction of GPR68 expression. (**A**) The binding of endogenous ARNTL or CLOCK to the *Gpr68* upstream region in Ly6G^−^/CD11b^+^/Ly6C^+^ cells prepared from *ARNTL* +/+ or *ARNTL* −/− Sham and 5/6Nx mice in the blood. The primer sets used are shown in Figure 2G. (**B**) The expression of *Gpr68* mRNA in Ly6G^−^/CD11b^+^/Ly6C^+^ cells prepared from *ARNTL* +/+ or *ARNTL* −/− Sham and 5/6Nx mice in the blood and spleen. (**C**,**D**) Flow cytometry analysis was performed to detect high-GPR68-expressing Ly6G^−^/CD11b^+^/Ly6C^+^ cells in the blood and spleen. The ratio of high-GPR68-expressing monocytes in the blood and spleen. For all panels, values are expressed as the mean ± S.D. (*n* = 5–7). **, *p* < 0.01, ** indicates significant differences between the two groups (two-way ANOVA with Tukey–Kramer post hoc tests).

Upregulated *Gpr68* and *Arntl* expression in the circulating blood monocytes of 5/6Nx mice alters circadian expression patterns [10]. Therefore, we analyzed monocytes in 5/6Nx mice lacking ARNTL in monocytes and macrophages. To investigate the relationship between ARNTL and GPR68 in vivo in 5/6Nx mice, we conducted the 5/6Nx procedure in monocytic ARNTL −/− mice, which were generated by crossing Lyz2Cre mice with *Arntl* loxp/loxp ICR mice (Figure S10). As reported in previous studies [40,41], ARNTL expression in monocytes and monocyte-derived cells from mice with both *Lyz2Cre* and *Arntl* loxp/loxp genes was completely abolished (Figure 1F). In addition, the loss of ARNTL abolished ARNTL binding to the upstream region of *Gpr68* in the circulating blood (Figure 3A). The level of CLOCK binding to the same region was also greatly suppressed (Figure 3A). These results suggested that the loss of ARNTL abolishes trans-activation by CLOCK/ARNTL upstream of *Gpr68*. According to previous studies [10,42,43], CD11b^+^ Ly6G^−^ Ly6C^+^ cells are mainly monocytes, and most GPR68-expressing cells in 5/6Nx hearts are derived from CD11b^+^ Ly6G^−^ Ly6C^+^ cells in the circulating blood or spleen [10]. Therefore, we measured *Gpr68* expression in CD11b^+^ Ly6G^−^ Ly6C^+^ cells from the spleen and circulating blood. *Gpr68* expression in monocytic ARNTL −/− 5/6Nx mice did not exceed that in Sham mice (Figure 3B). Monocytic ARNTL +/+ 5/6Nx mice expressed GPR68 in more than 90% of CD11b^+^ Ly6G^−^ Ly6C^+^ cells, while monocytic ARNTL −/− 5/6Nx mice had only a few GPR68-expressing CD11b^+^ Ly6G^−^ Ly6C^+^ cells (Figure 3C,D).

### 2.3. Effect of Loss of Monocyte-Specific ARNTL on CKD-Induced Cardiac Pathology Progression

**Figure 4:**
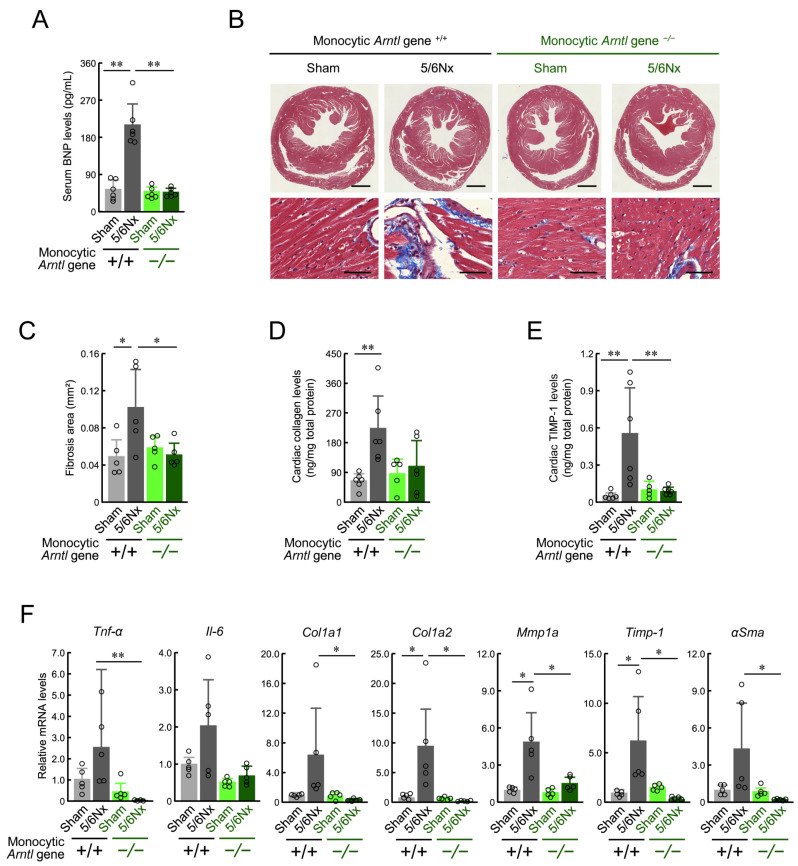
The effect of the deficiency of monocyte-specific ARNTL on 5/6Nx-induced cardiac injury. (**A**) Serum BNP concentrations in *ARNTL* +/+ or *ARNTL* −/− Sham and 5/6Nx mice. Values are expressed as the mean ± S.D. (*n* = 6). (**B**,**C**) Mutations in *Arntl* in monocytes ameliorated CKD-induced cardiac fibrosis. Panel (B) shows Masson’s trichrome staining of tissue fibrosis in blue. Scale bars indicate 1 mm (upper panel) and 50 μm (lower panel). Panel (**C**) shows the quantification of the fibrosis area under light microscopy. Values are expressed as the mean ± S.D. (*n* = 5). (**D**) The total amount of collagen throughout the ventricle. Values were corrected for total protein mass. Values are expressed as the mean ± S.D. (*n* = 5–6). (**E**) Cardiac TIMP-1 protein levels in *ARNTL* +/+ or *ARNTL* −/− Sham and 5/6Nx mice. Values were corrected for total protein mass. Values are expressed as the mean ± S.D. (*n* = 5–6). (**F**) The mRNA levels of *Tnf-α* and *Il-6* and fibrosis-related factors (*Col1a1*, *Col1a2*, *Mmp1a*, *Timp-1*, and *αSma*) in the cardiac ventricle of *ARNTL* +/+ or *ARNTL* −/− Sham and 5/6Nx mice. The mean value of the Sham-operated *ARNTL* +/+ group was set to 1.0. Values are expressed as the mean ± S.D. (*n* = 5). *, *p* < 0.05, **, *p* < 0.01 indicates significant differences between the two groups (two-way ANOVA with Tukey–Kramer post hoc tests).

When wild-type ICR mice were treated with 5/6Nx, elevated cardiac inflammation, fibrosis, and brain natriuretic peptide levels, which are indicators of heart failure, were observed eight weeks after surgery [10]. Therefore, we evaluated the cardiac pathology of monocytic ARNTL +/+ and ARNTL −/− mice. The changes in body weight (Figure S11A), water intake (Figure S11B), and food intake (Figure S11C) at eight weeks after the operation were similar to those in previous studies [31]; there was a slight decrease in body weight and an increase in water consumption due to 5/6Nx treatment. These changes were not influenced by ARNTL expression in monocytes. Notably, brain natriuretic peptide levels were increased in monocytic ARNTL +/+ 5/6Nx mice, but no such increase was observed in monocytic ARNTL −/− 5/6Nx mice (Figure 4A). In addition, the quantification of fibrotic areas in the heart revealed augmented fibrosis in monocytic ARNTL +/+ 5/6Nx mice but not in monocytic ARNTL −/− 5/6Nx mice (Figure 4B,C). Furthermore, the total amount of collagen and enzyme TIMP-1 protein, the synthesis of which increases as cardiac fibrosis progresses [44,45], in the ventricular tissue was elevated by 5/6Nx treatment in ARNTL +/+ mice but not in ARNTL −/− mice (Figure 4D,E). Similarly, the expression of the inflammatory cytokines TNF-α and IL-6 and the fibrosis markers Col1a1, Col1a2, Mmp1a, Timp-1, and αSma in the ventricles was elevated in monocytic ARNTL +/+ 5/6Nx mice but not in monocytic ARNTL −/− 5/6Nx mice (Figure 4F). These results suggest that the loss of ARNTL in monocytes suppressed the deterioration of cardiac pathology.

### 2.4. Effect of Loss of Monocyte-Specific ARNTL on CKD-Induced Renal Pathology Progression

**Figure 5:**
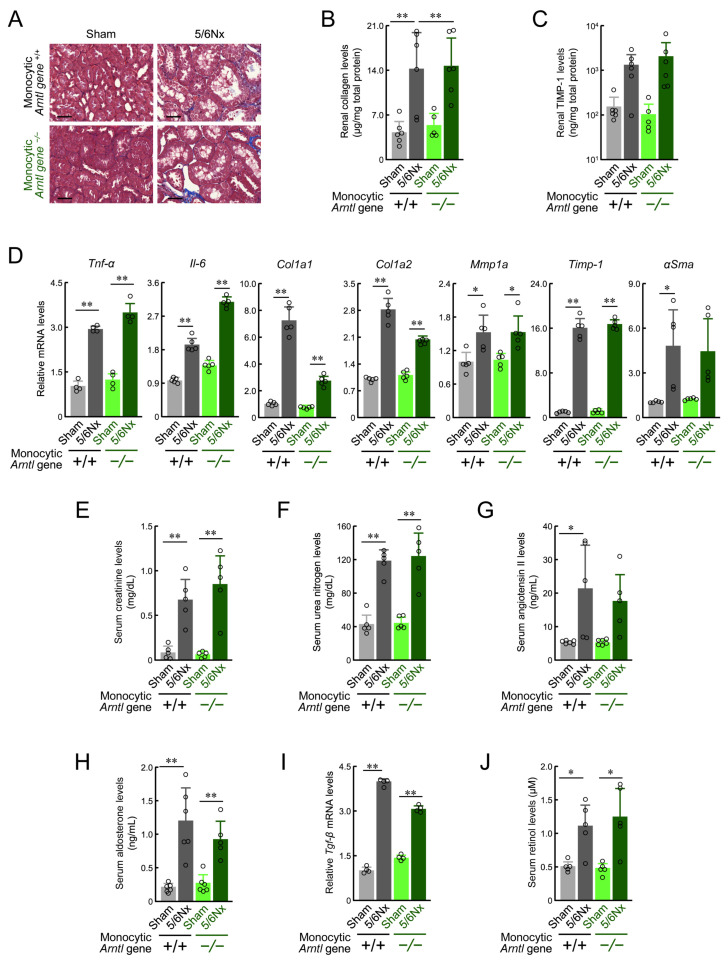
The effect of the loss of monocyte-specific ARNTL on renal function in 5/6Nx mice. (**A**) Masson’s trichrome staining for the kidneys prepared from *ARNTL* +/+ or *ARNTL* −/− Sham and 5/6Nx mice. Scale bars indicate 50 μm. (**B**) The total amount of collagen throughout the kidney. Values were corrected for total protein mass. Values are expressed as the mean ± S.D. (*n* = 5–6). (**C**) Renal TIMP-1 protein levels in *ARNTL* +/+ or *ARNTL* −/− Sham and 5/6Nx mice. Values were corrected for total protein mass. Values are expressed as the mean ± S.D. (*n* = 5–6). (**D**) The mRNA levels of *Tnf-α* and *Il-6* and fibrosis-related factors (*Col1a1*, *Col1a2*, *Mmp1a*, *Timp-1*, and *αSma*) in the kidney of *ARNTL* +/+ or *ARNTL* −/− Sham and 5/6Nx mice. The mean value of the Sham-operated *ARNTL* +/+ group was set to 1.0. Values are expressed as the mean ± S.D. (*n* = 5). (**E**–**H**) The serum concentrations of creatinine (**E**), urea nitrogen (**F**), angiotensin II (**G**), and aldosterone (**H**), in *ARNTL* +/+ or *ARNTL* −/− Sham and 5/6Nx mice. (**I**) The mRNA levels of *Tgf-β* in the kidney of *ARNTL* +/+ or *ARNTL* −/− Sham and 5/6Nx mice. The mean value of the Sham-operated *ARNTL* +/+ group was set as 1.0. (**J**) The serum concentrations of retinol in *ARNTL* +/+ or *ARNTL* −/− Sham and 5/6Nx mice. In all panels, values are expressed as the mean ± S.D. (*n* = 4–5). **, *p* < 0.01; *, *p* < 0.05 significant difference between the two groups (one-way or two-way ANOVA with Tukey–Kramer post hoc tests).

Wild-type ICR 5/6Nx mice showed elevated blood angiotensin II, aldosterone, and urea nitrogen levels associated with kidney damage eight weeks after surgery [10,31,32,33]. In contrast to cardiac pathology, no obvious difference was seen in terms of kidney tissue damage with or without ARNTL (Figure 5A). The 5/6Nx-induced increase in collagen and TIMP-1 protein content in the kidney and the expression of a group of genes indicative of fibrosis progression were also unaffected by the presence or absence of ARNTL expression (Figure 5B–D). In addition, the levels of angiotensin II, aldosterone, creatinine, and urea nitrogen in the blood were increased by the 5/6Nx procedure in both monocytic ARNTL +/+ and monocytic ARNTL −/− mice (Figure 5E–H). Similarly, no effect of monocyte ARNTL loss was observed for increased renal *Tgfb* expression or increased blood retinol levels, which are associated with the degree and progression of kidney damage (Figure 5I,J). These results indicate that the loss of ARNTL in monocytes did not inhibit the 5/6Nx-induced deterioration of renal pathology. Furthermore, they indicate that the progression of heart pathology due to the loss of ARNTL in monocytes was not because of improved renal function or indirectly via blood molecules such as angiotensin II and aldosterone, which can cause cytotoxicity and increased cardiac load, but rather due to ARNTL-deficient cells infiltrating the heart.

### 2.5. Effect of Loss of Monocyte-Specific ARNTL on Expression of Membrane Molecules Indicative of Function of Cardiac Macrophages in 5/6Nx Mice

**Figure 6:**
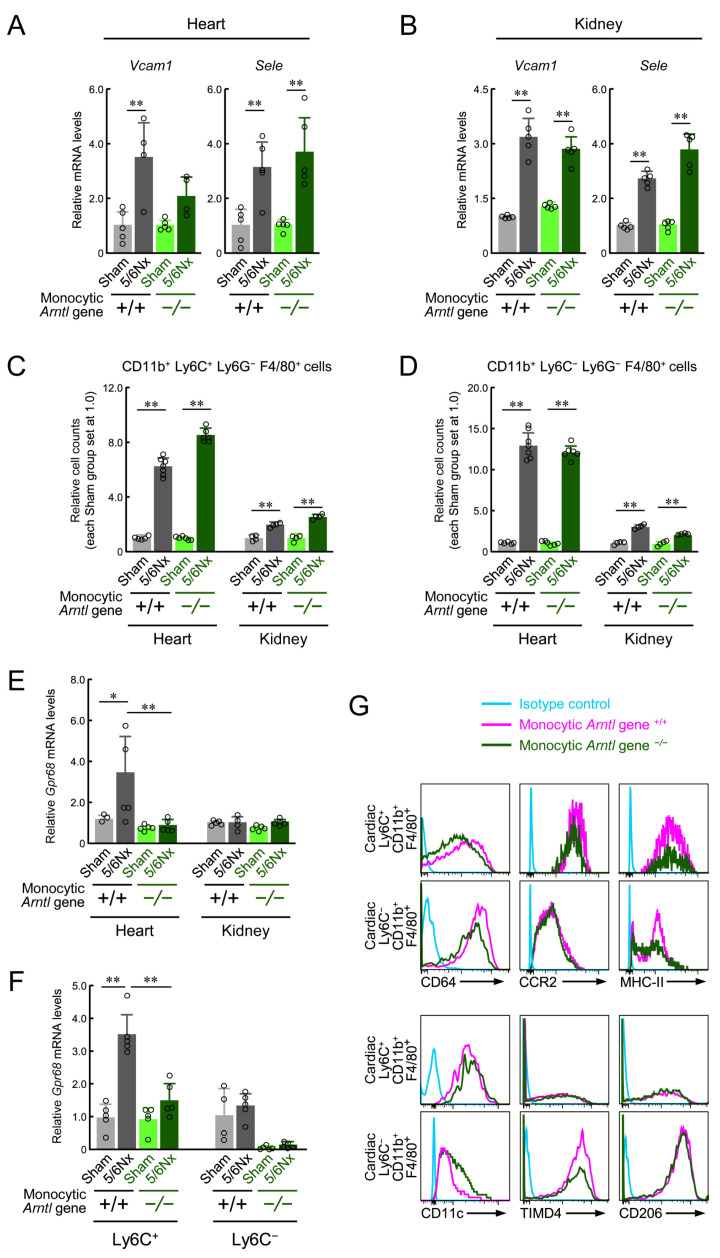
The effect of the loss of monocyte-specific ARNTL on the 5/6Nx-induced induction of GPR68 expression. (**A**,**B**) The mRNA levels of *Vcam1* and *Sele* in the cardiac ventricle or kidney of *ARNTL* +/+ or *ARNTL* −/− Sham and 5/6Nx mice. The mean value of the Sham-operated *ARNTL* +/+ group was set to 1.0. (**C**,**D**) The number of cardiac or renal F4/80^+^/Ly6G^−^/CD11b^+^/Ly6C^+^ cells (**C**) and F4/80^+^/Ly6G^−^/CD11b^+^/Ly6C^−^ cells (**D**) in each organ. The mean value of the Sham-operated *ARNTL* +/+ group in each organ was set as 1.0. (**E**) The mRNA levels of *Gpr68* in the cardiac ventricle or kidney of *ARNTL* +/+ or *ARNTL* −/− Sham and 5/6Nx mice. The mean value of the Sham-operated *ARNTL* +/+ group was set to 1.0. (**F**) The expression of *Gpr68* mRNA in cardiac F4/80^+^/Ly6G^−^/CD11b^+^/Ly6C^+^ and F4/80^+^/Ly6G^−^/CD11b^+^/Ly6C^−^ cells prepared from *ARNTL* +/+ or *ARNTL* −/− Sham and 5/6Nx mice ventricles. (**G**) The expression levels in the cardiac F4/80^+^/Ly6G^−^/CD11b^+^/Ly6C^+^ and F4/80^+^/Ly6G^−^/CD11b^+^/Ly6C^−^ cells of markers indicative of a subset of macrophages. Histograms showing the expression of each marker were obtained by flow cytometric analysis. For all panels, values are expressed as the mean ± S.D. (*n* = 4–7). *, *p* < 0.05, **, *p* < 0.01 indicates significant differences between the two groups (two-way ANOVA with Tukey–Kramer post hoc tests).

We investigated the causes underlying the differential effects of the loss of monocyte-specific ARNTL on 5/6Nx-induced fibrosis outcomes in the kidneys and heart. The loss of monocytic ARNTL did not affect the increase in adhesion molecules VCAM1 and SELE (Figure 6A,B) nor the increase in the number of monocytes, granulocytes, and macrophages in both heart and kidney tissues after 5/6Nx treatment (Figure 6C,D and Figure S12A,B). On the other hand, when these increases were compared between the heart and kidneys, differences were found in the increase in monocyte and macrophage abundance, despite the lack of differences regarding the increased expression of adhesion molecules by 5/6Nx treatment. *Gpr68* expression in the kidneys was not considerably affected by 5/6Nx or ARNTL (Figure 6E). In contrast, *Gpr68* expression in the heart was significantly affected by the ARNTL defect, and the 5/6Nx-induced increase in *Gpr68* expression observed in ARNTL +/+ mice was not observed in ARNTL −/− mice (*p* < 0.05; Figure 6E).

Subsequently, we further investigated GPR68 expression and subtypes of cardiac macrophages. Cells highly expressing GPR68 in the heart are characterized by F4/80^+^ CD11b^+^ Ly6G^−^ Ly6C^+^ [10]. The number of F4/80^+^ CD11b^+^ Ly6G^−^ Ly6C^+^ cells in the heart was increased by 5/6Nx treatment, and this increase was not influenced by the loss of monocyte ARNTL (Figure 6C). *Gpr68* expression in F4/80^+^ CD11b^+^ Ly6G^−^ Ly6C^+^ cells was affected by the ARNTL defect, and the 5/6Nx-induced increase in the *Gpr68* expression observed in ARNTL +/+ mice was not observed in ARNTL −/− mice (Figure 6F). To investigate the F4/80^+^ CD11b^+^ Ly6G^−^ Ly6C^+^ cell subset in detail, we performed further flow cytometry analyses to assess the expression of cell membrane molecules (CD64, CCR2, MHC-II, CD11c, TIMD4, and CD206) associated with tissue macrophages [43,46,47,48,49,50]. Similar to cells derived from ARNTL +/+ 5/6Nx and ARNTL −/− 5/6Nx mice, the majority of the cells expressed CD64; highly expressed CCR2, MHC-II, CD11c, and TIMD4; and did not express CD206 (Figure 6G). In addition, the cells had a high level of expression of *Il1b* and a low level of expression of *Lyve1* and *Folr2* mRNA (Figure S13). The expression of these markers indicated that the cells were pro-inflammatory macrophages and that the majority were derived from monocytes [43,46,47,48,49,50]. The same investigation was performed on Ly6C-negative cardiac macrophages (F4/80^+^ CD11b^+^ Ly6G^−^ Ly6C^−^ cells). As with Ly6C-positive cells, the loss of ARNTL did not affect the increase in cell number caused by 5/6Nx treatment (Figure 6D). In contrast with that in Ly6C-positive cells, *Gpr68* expression in Ly6C-negative cells was not increased by the 5/6Nx procedure in ARNTL +/+ mice (Figure 6F). The expression of marker molecules indicating subsets was as follows: CD64, positive; CCR2 and MHC-II, mixed positive and negative; CD11c, low expression; TIMD4, positive; CD206, positive; and *Lyve1* and *Folr2* mRNA, positive. This indicated that the cells were primarily anti-inflammatory macrophages and contained both yolk sac macrophages and fetal monocyte-derived cells (Figure 6G and Figure S13). In addition, the expression of these marker molecules was not affected by the loss of ARNTL. These results showed that the cardiac cells with upregulated GPR68 expression after 5/6Nx treatment were primarily of monocyte origin and that GPR68 expression was affected by the loss of monocyte ARNTL, but a subset of cardiac macrophages was not affected by this loss.

In summary, our research revealed the role of ARNTL in monocytes in vivo with reduced renal function through a detailed analysis of the loss of monocyte-specific ARNTL and heart and kidney damage. As there are few previous studies that have combined cell-specific genetic manipulation and nephrectomy, one of the strengths of this study is that it analyzed in detail the relationship between monocyte ARNTL and the state in which the nephrons were highly lost. Another strength of this study is that it focused on monocyte GPR68, which we discovered in our previous study [10,25], to analyze the differences between the kidneys and hearts in the 5/6Nx condition. However, since actual patients with CKD lose nephrons due to various causes, including diabetes, it is difficult to immediately extrapolate our research results to medical practice. In order to achieve this, it will probably be necessary to accumulate similar verification results for other mouse models that exhibit CKD, such as adenine-loaded, hypertensive, and diabetic model mice.

## 3. Discussion

Optimized prevention methods are urgently needed for CKD and related cardiovascular disorders, due to the immense global health challenge they pose [51]. Circadian clock mechanisms have been associated with the onset and progression of CKD and cardiovascular disorders [52,53,54]. This study demonstrates that the loss of ARNTL in monocytes suppresses 5/6Nx-induced cardiac injury. The findings observed in wild-type 5/6Nx mice are similar to those observed in patients with preserved ejection fraction (HFpEF) [10,55]. HFpEF is prevalent in approximately half of all patients with heart failure [56,57], and its prognosis is poorer than that of heart failure with reduced ejection fraction, owing to a lack of established treatment. Our findings may elucidate and provide a reference for potential treatment options for HFpEF. Inflammation and chronic inflammatory processes involving monocytes have been implicated in the pathogenesis of heart failure [58,59]. Notably, patients with HFpEF exhibit mild chronic systemic inflammation, as evidenced by higher levels of inflammatory cytokines in their blood and increased immune cell infiltration in cardiac tissue [58]. Furthermore, CKD is a prominent risk factor for systemic chronic inflammation, as patients with CKD exhibit higher levels of inflammatory cytokines in their blood and increased monocyte infiltration in cardiac tissue [23,60,61]. Cohort studies have highlighted the correlation between this mild chronic inflammatory state and a poor prognosis [60], as serum TNFα and IL-6 levels in patients with CKD are appreciably higher than those in healthy controls [10,62]. The ARNTL-mediated induction of monocyte GPR68 expression may be involved in systemic inflammation, serving as a potential risk factor for cardiac disease during CKD.

Notably, leukocyte counts in peripheral blood and the production of inflammatory cytokines exhibit distinct circadian rhythms, which form part of the body’s protective mechanism against infections and other risks. Recent evidence highlights the expression of clock genes in leukocytes, including monocytes, and their influence on regulating cellular functions. Clock genes in leukocytes have been implicated in various diseases. Clock gene abnormalities in T cells and monocytes resulting from hypocortisolemia have been associated with the exacerbation of the disease state. Conversely, the restoration of the expression rhythm of clock genes in T cells and monocytes has been shown to improve the disease state [63]. In this study, we investigated the functionality of the circadian clock mechanism in RAW264.7 and THP-1 cells. Our findings demonstrated that DEX treatment synchronized the rhythm of clock gene expression in monocytes as well as the circadian rhythm of GPR68 expression. These results suggest that an endogenous circadian clock mechanism can rhythmically regulate GPR68 expression in monocytes and macrophages. Clock genes in monocytes may play an important role in biological defense by generating circadian rhythms in infection and innate immune responses via GPR68.

GPR68 is highly expressed in microvascular endothelial cells and monocytes/macrophages [64]. As a unique GPCR activated by protons and shear stress [65,66], it functions in sites of necrosis and inflammation where pH reduction occurs and in vessels under shear stress. In monocytes/macrophages, ARNTLs regulate the expression of PKM2 and S100A9, influencing their function via the glycolytic system [67,68]. This study reveals a correlation between ARNTL deficiency, GPR68 expression, and cardiac pathology in CKD. The results indicate that the role of cardiac macrophages in CKD-associated cardiac pathology is highly dependent on the expression of GPR68, which is, in turn, regulated by ARNTL. A plausible explanation for this observation is that the constant beating of the heart renders it susceptible to shear stress, making GPR68 easily activated in this environment. In addition, GPR68 expression decreases in environments with high concentrations of TGF-β, such as the kidneys of 5/6Nx mice [10]. Moreover, our study demonstrates that the loss of ARNTL in monocytes does not suppress the elevated levels of angiotensin II or urea nitrogen, which are indicative of renal injury, suggesting that the role of GPR68-mediated ARNTL is highly dependent on the tissue environment, even in the same disease state in vivo. Although drawing definitive conclusions from this study may be challenging, the development of a method for the in vivo determination of GPR68 activity could provide a further validation of these considerations.

Our results showing that PU.1 was required for the induction of GPR68 expression by ARNTL suggests that 5/6Nx-induced GPR68 expression is a monocyte/macrophage-specific phenomenon. In addition, as shown in previous studies [10,42,43] and Figure 3, elevated GPR68 expression induced by 5/6Nx treatment was observed in both CD11b^+^ Ly6G^−^ Ly6C^+^ cells from in the circulating blood and spleen, as well as F4/80^+^ CD11b^+^ Ly6G^−^ Ly6C^+^ cells present in the heart. Furthermore, this cell population in the heart primarily comprised cells that expressed CCR2 and MHC-II and did not express TIMD4, LYVE1, or FOLR2, suggesting that these cells were derived primarily from monocytes [43,46,47,48,49,50]. The loss of monocytic ARNTL did not affect the expression of these markers or CD11c, the marker of pro-inflammatory cells (Figure 6G). This is consistent with the results of a previous study reporting that the loss of ARNTL does not affect lipopolysaccharide-induced differentiation into inflammatory macrophages [27]. In other words, ARNTL is suggested to make few contributions to the differentiation of bone marrow-derived cells in 5/6Nx mice. In contrast to cardiac Ly6C-positive cells, the fact that the *Gpr68* expression in cardiac Ly6C-negative cells was not upregulated by 5/6Nx treatment may be related to the fact that this cell population expressed high levels of TIMD4, CD206, LYVE1, and FOLR2 (Figure 6F,G and Figure S13). Because the expression of these markers suggests that this cell population contains yolk sac-derived cells and is anti-inflammatory [43,46,47,48,49,50], the origin and function of such cells may contribute to GPR68 expression. It is difficult to conclude the cause of the difference from this study, but a more detailed analysis focusing on the cellular origin and STRA6, which triggers *Gpr68* upregulation, could elucidate the cause of this phenomenon.

The loss of monocytic ARNTL suppressed 5/6Nx-induced cardiac fibrosis but not renal dysfunction, indicating drastic differences between the outcomes of the two organs due to 5/6Nx treatment. There are numerous similarities between the progression of cardiac and renal fibrosis. In both, damage to cardiomyocytes and glomeruli induces the expression of adhesion molecules such as E-selectin, VCAM1, and ICAM1 in endothelial cells via transcription factors such as NF-κB, thereby promoting the infiltration of immune cells such as granulocytes, monocytes, and macrophages [45,69,70]. These infiltrating immune cells then produce cytokines and chemokines and clear debris, thereby regulating the inflammatory response and activating myofibroblasts and fibroblasts, which, in turn, leads to the formation of the extracellular matrix (ECM) and microvascular network [45,69,70]. Additionally, 5/6Nx treatment induces the expression of E-selectin and VCAM1 in both the heart and kidney and increases the number of monocytes, granulocytes, and macrophages in tissues [10]. However, neither retinol, which upregulates ARNTL expression under CKD, nor GPR68 expression affects these elevations [10]. In addition, the loss of monocytic ARNTL did not affect the increase in *Vcam1* and *Sele* expression (Figure 6A,B) or the number of monocytes, granulocytes, and macrophages in heart and kidney tissues caused by 5/6Nx treatment (Figure 6C,D and Figure S12A,B). This suggests that monocytic ARNTL is commonly involved in the immune cell infiltration of both the heart and kidneys but is involved in the formation of the ECM and microvascular network, the cause of fibrosis, only in the heart. Because GPR68 expression was not upregulated and few GPR68-expressing cells were found in the kidneys of 5/6Nx mice (Figure 6E) [10], the differential role of monocytic ARNTL across tissues is likely related to GPR68 expression. Because the expression of *Mmp1a* and *Timp-1*, enzymes involved in collagen metabolism, was altered (Figure 4F), cardiac macrophages may increase the activity of fibroblasts and myofibroblasts, which are responsible for the synthesis of these enzymes and the formation of the ECM via GPR68, leading to progressive fibrosis.

One possible reason for the absence of GPR68-expressing cells in the kidneys of 5/6Nx mice is the amount of TGFβ present; the kidneys of 5/6Nx mice are highly loaded immediately after surgery by surgically removing most of the kidney tissue. This leads to an increase in immune cells and fibroblasts, which have the ability to produce TGF-β, with concomitant ECM formation and collagen accumulation. In fact, marked increases in these parameters are observed before cardiac fibrosis occurs [10,31,33], and the kidneys of 5/6Nx mice express approximately 20 times more TGF-β1 than the heart [10]. Furthermore, TGF-β exposure suppresses GPR68 expression [10]; these phenomena may explain why TGF-β1 downregulates *Gpr68* expression in the renal macrophages of 5/6Nx mice. The differences in the function of the ECM and cells that form the tissue environment, such as fibroblasts, may determine the ARNTL- or GPR68-mediated functions of monocytes after infiltration. Differences in the timing of damage to each organ and the timing of retinol accumulation may also be associated with this difference in outcomes. Notably, 5/6Nx mice exhibited elevated creatinine and angiotensin II levels in the blood and TGF-β1 in the kidney within two weeks of surgery [33]. This indicates that damage occurs in the kidneys during this period; therefore, many monocytes can infiltrate the kidney at this time. In contrast, elevated blood retinol and associated elevated GPR68 expression levels were not observed until four weeks after surgery, and GPR68-high monocytes in the blood did not appear until later [10,33]. In other words, monocytes infiltrating the kidney immediately after surgery through the onset of failure at week 2 did not express high levels of GPR68 at that time, which may also be an underlying reason for the differences in outcomes between the organs demonstrated in this study. Another possible cause could be the difference in the blood flow connections to the spleen between various organs. During heart injury, monocytes stored in the spleen migrate to the heart in large numbers through the splenic vein [42], and more than 90% of the GPR68-high-expressing cells that migrate to the heart originate from the spleen in 5/6Nx mice [10]. Blood flow originating from the splenic vein reaches the heart via the portal vein and the kidneys via the aorta. In simpler terms, the blood containing GPR68-expressing monocytes released from the spleen cannot reach the kidneys without passing through the heart. In addition, blood flow to the spleen increases during heart failure, while blood flow to the kidneys decreases [71]. Upon comparing the rate of increase in the number of relevant cells in the heart and kidneys at eight weeks after surgery, the macrophage, monocyte, and granulocyte levels in the hearts of 5/6Nx mice with and without ARNTL were all found to be approximately 10–15 times higher than those in the hearts of Sham mice but only 2–3 times higher in the kidneys of 5/6Nx mice than those in the kidneys of Sham mice (Figure 6C,D and Figure S12A,B). This suggests that the kidneys are less accessible to GPR68-expressing monocytes than the heart, possibly explaining why renal macrophages in 5/6Nx mice do not express elevated levels of GPR68 and why renal injury caused by 5/6Nx is not dependent on GPR68.

Several studies have pointed out that there is a close relationship between CKD, heart failure, and clock genes [72,73,74]. However, the fact that clock genes independently affect diabetes [75], obesity [75], and hypertension [76,77], which are risk factors for CKD and heart failure, and that clock genes play different roles in different cells such as immune cells [78,79], vascular smooth muscle cells [80], fibroblasts [81], and adipocytes [82], is a major barrier to analyzing the relationship between CKD, heart failure, and clock genes using molecular biology. In addition, the induction of renal damage caused by the administration of adenine [13], which is often used for CKD mouse models, causes damage to the renal tubules prior to the loss of nephrons. This may differ from the pathology of patients with CKD, in which the loss of nephrons due to an increase in the intraglomerular pressure often precedes the onset of the disease. The two methods we used in our present study—the loss of monocyte/macrophage-specific ARNTL molecules and the surgical removal of nephrons by 5/6 nephrectomy—provide new evidence linking the relationship between cardiac/renal impairment associated with chronic reductions in the glomerular filtration rate and ARNTL in monocytes/macrophages at the molecular biological level. In order for humanity to overcome CKD and heart disease, it is of course extremely important to treat diabetes and hypertension, which account for the majority of the causes of CKD, but it may also be necessary to conduct detailed analyses of the causal molecules that cause heart impairment due to CKD for each factor and cell, as in our present study, and to construct a method of dealing with it based on the causal relationship.

## 4. Materials and Methods

### 4.1. Animals and Treatments

Lyz2Cre ICR mice with Cre knock-in at the lysozyme 2 locus and *Arntl* loxp/loxp ICR mice with loxp sequences inserted at the *Arntl* locus were generated by crossing B6.129P2-Lyz2<tm1(cre)Ifo>/J (Jackson Laboratory, Bar Harbor, ME, USA) and B6.129S4(Cg)-ARNTL<tm1Weit>/J (Jackson Laboratory) mice with Jcl:ICR mice for more than nine generations. Among the assortments obtained by crossing the Lyz2Cre ICR mice with ARNTL loxp/loxp ICR mice for three generations, male mice carrying only *Arntl* loxp/loxp were designated as monocyte ARNTL-expressing (ARNTL +/+) mice, and those carrying *Arntl* loxp/loxp and Lyz2Cre were used for experiments as monocyte-specific ARNTL-deficient (ARNTL −/−) mice. Male ICR mice were paired based on sex (male), weight, and age and randomly selected for experiments. Mice were housed in a light-controlled room with a light/dark cycle from ZT0 to ZT12 at a temperature of 24 ± 1 °C and humidity of 60 ± 10%, with free access to water and a normal pelleted diet. The mice were synchronized with a light/dark cycle for two weeks before surgery. Then, the 5/6Nx procedure was performed as described in previous studies [10,31,32,33]. CKD mouse models were generated using a two-step 5/6Nx model at five or six weeks of age. During the first surgery, two-thirds of the left kidney was removed by cutting off both poles. Then, seven days later, the right kidney was excised. The mice were housed for eight weeks after the operation until CKD developed. Sham mice underwent laparotomies on the same day the experimental mice underwent 5/6Nx surgery (Figure S10). The data showing the renal function of each mouse after surgery are shown in Section 2.

### 4.2. Cell Culture and Treatment

RAW264.7 mouse macrophage-like cells (RRID: CVCL_0493) and THP-1 human monocyte-like cells (RRID: CVCL_0006) were purchased from the American Type Culture Collection (Manassas, VA, USA). NIH3T3 fibroblasts (RRID: CVCL_0594) were purchased from the Cell Resource Center for Biomedical Research (Tohoku University, Sendai, Japan). The RAW264.7 and NIH3T3 cells were cultured in Dulbecco’s modified Eagle’s medium supplemented with 5% fetal bovine serum (FBS) and 0.5% penicillin–streptomycin solution (Invitrogen; Life Technologies, Carlsbad, CA, USA) and maintained at 37 °C in a humidified 5% CO_2_ atmosphere. Primary mouse monocytes were collected from the blood of 6-week-old ICR mice using a MACS Monocyte Isolation Kit (Miltenyi Biotec Ltd., Bisley, UK). THP-1 and mouse primary monocytes were cultured in RPMI 1640 medium supplemented with 0.5% penicillin–streptomycin solution and 10% mouse serum under a 5% CO_2_ environment at 37 °C. To synchronize the circadian clock, RAW264.7 cells were exposed to 100 nmol/L DEX [35,79]. After 2 h, the medium and culture conditions were returned to those prior to DEX exposure (5% FBS, 0.5% penicillin–streptomycin, 37 °C, 5% CO_2_). *Arntl*-KO RAW264.7 cells were prepared using the CRISPR/Cas9 system. Cells were transfected with *ARNTL* CRISPR/Cas9 and *ARNTL* HDR plasmids (Santa Cruz Biotechnology, Inc., Dallas, TX, USA), and after incubation for 24 h, *ARNTL*-KO cells were selected using 10 g/mL puromycin. The medium was replaced with puromycin every few days for two weeks, after which *ARNTL*-knockout cells were isolated. *Stra6* siRNA (Thermo Fisher Scientific, Waltham, MA, USA) and *Cebpa* miRNA (Thermo Fisher Scientific) were transfected into RAW264.7 cells using Lipofectamine™ LTX and PLUS™ reagents (Thermo Fisher Scientific). The siRNA oligonucleotide sequences were as follows: *Stra6* siRNA sense 5′-GCUGCUGUCUUUGUGGUCCUCUUCA-3′ and antisense 5′-UGAAGAGGACCACAAAGA-CAGCAGC-3′ and control siRNA sense 5′-GCUGUCUUUGUGUUGCCCUUCGUCA-3′ and antisense 5′-UGACGAAGGGCAACACAAAGACAGC-3′.

### 4.3. Construction of Gpr68 or Arntl Reporter Plasmid-Expressing RAW264.7 Cells

The 5′-flanking region of mouse *Gpr68* and *Arntl* was amplified using Elongase Enzyme mix (Invitrogen). The distance from the transcription start point of the region and primer sequences are shown in Table S1. These PCR products were ligated into pGL4.18 using the Ligation-Convenience Kit (NIPPON GENE Co., Ltd., Tokyo, Japan). These plasmids were transfected into RAW264.7 cells using Lipofectamine™ LTX and PLUS™ reagents (Thermo Fisher Scientific).

### 4.4. Construction of RAW264.7 Cells Stably Expressing ARNTL

Mouse ARNTL-expressing plasmid (MR209553, OriGene Technologies, Inc., Rockville, MD, USA) was transfected into RAW264.7 cells using Lipofectamine™ LTX and PLUS™ reagents (Thermo Fisher Scientific). Cells stably expressing ARNTL were selected using 4000 µg/mL G418 (FUJIFILM, Tokyo, Japan), and individual colonies were subsequently expanded and maintained in media containing 4000 µg/mL G418.

### 4.5. Construction of PU.1-Expressing NIH3T3 Cells

Mouse PU.1-expressing plasmid (MR203632, OriGene) was transfected into NIH3T3 cells using Lipofectamine™ LTX and PLUS™ reagents (Thermo Fisher Scientific). PU.1-expressing cells were selected using 4000 µg/mL G418 (FUJIFILM), and individual colonies were subsequently expanded and maintained in media containing 4000 µg/mL G418.

### 4.6. Quantitative RT-PCR

The QIAGEN RNeasy Mini kit (QIAGEN, Hilden, Germany) was used for RNA extraction and a ReverTra Ace™ qPCR RT kit (Toyobo, Osaka, Japan) for cDNA synthesis by reverse transcription. RT-PCR analysis was performed using THUNDERBIRD™ SYBR qPCR Mix (Toyobo) and a 7500 RT-PCR system (Applied Biosystems, Foster City, CA, USA). Data were normalized using Actb. Primer sequences are shown in Table S2.

### 4.7. Western Blotting

Protein samples from cells or heart tissue were prepared using CelLytic™ MT Cell Lysis Reagent (Merck KGaA, Darmstadt, Germany). After separation using SDS-PAGE and transferring to Immobilon-P (Merck Millipore, Marlborough, MA, USA), the membranes were exposed to primary antibodies against GPR68 (1:1000; CSB-PA060199, CUSABIO, Houston, TX, USA), ARNTL (1:1000; ab-93806, Abcam, Cambridge, UK), STRA6 (1:1000; NBP1-00242, Novus Biologicals, Centennial, CO, USA), ACTB (1:1000; SC-47778, Santa Cruz Biotechnology), and PU.1 (1:1000; ab76543, Abcam) diluted using the Can Get Signal Immunoreaction Enhancer Solution (Toyobo). Afterward, the membranes were incubated with anti-rabbit IgG secondary antibodies, followed by detection using Chemi-Lumi One reagent (Nacalai Tesque, Inc., Kyoto, Japan). Density was analyzed using an ImageQuant LAS 3000 mini instrument (FUJIFILM).

### 4.8. Luciferase Reporter Assay

Cells were seeded onto 24-well culture plates at a density of 2 × 10^5^ cells per well. The cells were then transfected with *Clock*- and *Arntl*-expressing plasmids. To ensure the same amount of DNA in all transfections, the pcDNA3.1 empty vector was added. In addition, as an internal control reporter, 10 ng of the phRL-TK vector (Promega, Madison, WI, USA) was co-transfected. After 24 h, the cells were harvested, and the lysates were analyzed using the Dual-Luciferase^®^ Reporter Assay System (Promega). The normalized luciferase activity was determined by calculating the firefly-to-luciferase activity ratio in each sample.

### 4.9. Histochemical Staining

Masson’s trichrome (MT) staining was performed according to the procedure outlined in previous studies [10,31,32,33]. Removed hearts were placed in 4% paraformaldehyde in phosphate-buffered saline. After 12 h, the heart tissues were paraffin-embedded and stained with MT. Quantification and analysis were performed using a BZ-9000 fluorescence microscope (KEYENCE CORPORATION, Osaka, Japan). Fibrotic areas in the heart were quantified based on the blue areas of the entire cross-section of the MT-stained ventricular sections after the heart was sliced in a circle from the direction where both the right and left ventricles were visible. The hue of the blue-stained fibrotic areas in the image, including the entire ventricle (TIFF format), taken with a Keyence BZ-9000 fluorescence microscope at 20× objective magnification, was specified using Hybrid Cell Count (BZ-H3C; KEYENCE CORPORATION), and the areas were quantified using the same software.

### 4.10. Quantification Blood, Kidney, and Heart Factors

The serum angiotensin II level was measured using an angiotensin II ELISA kit (Enzo Life Sciences, Farmingdale, NY, USA). The serum aldosterone level was measured using an aldosterone ELISA kit (Enzo Life Sciences). The serum brain natriuretic peptide level was measured using a brain natriuretic peptide ELISA Kit (RayBiotech, Shanghai, China). Serum blood urea nitrogen levels were measured using a QuantiChrom Urea Assay Kit (Bioassay Systems, Hayward, CA, USA). Cardiac and renal TIMP-1 levels were measured using a Mouse TIMP-1 Quantikine ELISA Kit (R & D Systems, Minneapolis, MN, USA). Cardiac collagen levels were measured using a Total Collagen Assay Kit (QuickZyme Biosciences, Leiden, The Netherlands).

### 4.11. Flow Cytometry Isolation and Analysis

The isolation of cells for flow cytometry was performed as previously described [10]. The ventricles were digested in phosphate-buffered saline containing 500 μg/mL collagenase type II (FUJIFILM), 200 μg/mL CaCl_2_ (Nacalai Tesque), 0.05% trypsin (Sigma-Aldrich, St. Louis, MO, USA), and 10% FBS at 37 °C for 10 min with agitation. After treatment with RBC Lysis buffer (BioLegend, San Diego, CA, USA) and mouse TruStain FcX^TM^ (BL-101320, BioLegend), samples were stained with anti-F4/80-PerCP (BL-123108, BioLegend), anti-CD11b-PE (BL-101208, BioLegend), anti-Ly6C-APC (BL-128016, BioLegend), anti-GPR68 (CSB-PA060199, CUSABIO), anti-CD11c-FITC (BL-117305, BioLegend), anti-CCR2-FITC (BL-150607, BioLegend), anti-MERTK-FITC (BL-151503, BioLegend), anti-H-2-FITC (BL-125507, BioLegend), anti-Tim-4-PE (BL-130005, BioLegend), anti-CD64-PE (BL-130005, BioLegend), and/or anti-rabbit IgG-AF647 (ab-150107, Abcam). Dead cells were labeled using eFluor 780 viability dye (BD-565388, BD Biosciences, Erembodegem, Belgium). Quantification and analysis were performed using flow cytometry (Aria III; BD Biosciences, Franklin Lakes, NJ, USA) and FlowJo (Tree Star, Inc., San Carlos, CA, USA). The gating strategy is shown in Figure S14.

### 4.12. ChIP Analysis

ChIP experiments were performed as described in a previous study [10]. Chromatin samples conditioned from the cells were incubated with antibodies against ARNTL (ab-93806, Abcam), CLOCK (ab-3517, Abcam), or rabbit IgG (MEDICAL & BIOLOGICAL LABORATORIES Co., Ltd., Tokyo, Japan). DNA was purified using a QIAquick PCR purification kit (Qiagen) and amplified using PCR for E-box-binding elements in the 5′-flanking region of the mouse *Gpr68* gene. Primer sequences for amplification are shown in Table S2. THUNDERBIRD SYBR qPCR Mix (Toyobo) was used with the 7500 real-time PCR system (Applied Biosystems) to quantify the products. All data were normalized to the PCR products of input DNA. The quantitative reliability of PCR was evaluated using a kinetic analysis of the amplified products to ensure that signals were only from the exponential phase of amplification. This analysis was performed in the absence of an antibody or in the presence of rabbit IgG as a negative control. Ethidium bromide staining showed no PCR products in these samples.

### 4.13. Measurement of Retinol Concentrations

The measurement of retinol concentrations was performed as previously described [10]. Retinol was resolved using an AQUITY UPLC HSS PFP column (50 mm × 2.1 mm, 50 mm, p/n186005965; Waters, Milford, MA, USA) on an AQUITY UPLC H-Class XHCLQT0100 system (Waters). The multiple-reaction monitor was set at a mass-to-charge ratio (*m*/*z*) of 286.4–92.9 *m*/*z* for retinol and 329.1–151.1 *m*/*z* for the internal standard.

### 4.14. Data and Statistical Analysis

All statistical analyses were performed using the JMP^®^ Pro 13.0.0 (SAS Institute Japan, Tokyo, Japan). Differences were analyzed using a one- or two-way ANOVA and the Tukey–Kramer test among multiple groups and Student’s *t*-test between pairs of groups. All statistical tests were two-sided, and statistical significance was set at *p* < 0.05. In this study, “*n*” refers to the number of animals, with one dataset per mouse for all mice. The sample size was not determined using a specific statistical method but is comparable to those reported in previous publications [10,83,84]. All data presented in this manuscript, including Western blot photographs and microscopic images, were confirmed for reproducibility using at least three independent experiments.

## 5. Conclusions

Our study reveals that GPR68 expression in monocytes/macrophages is dependent on the expression of ARNTL, a central component of the circadian clock mechanism. The loss of monocyte-specific ARNTL is associated with altered GPR68 expression and suppresses 5/6Nx-induced cardiac damage. Because the expression of GPR68 and ARNTL in monocytes in patients with CKD correlates with renal function [10], targeting these molecules in monocytes/macrophages could offer potential therapeutic strategies for treating CKD-induced cardiovascular disorders. In addition, this study underscores the significance of investigating the function of GPR68, the expression of which is regulated by ARNTL, to understand the role of ARNTLs in monocytes/macrophages in diseases other than CKD.

## Data Availability

All data supporting the results of the present study presented in the study are presented in this article/Supplementary Materials. Further inquiries can be directed to the corresponding author.

## Supplementary Materials

The following supporting information can be downloaded at https://www.mdpi.com/article/10.3390/ijms252313009/s1.
