# Supplementary material for: Monocyte/Macrophage-Specific Loss of ARNTL Suppresses Chronic Kidney Disease-Associated Cardiac Impairment
Source: Int J Mol Sci. 2024 Dec 3;25(23):13009. doi: 10.3390/ijms252313009 (PMC11641442; doi:10.3390/ijms252313009)
Supplement: Supplementary file 1 [file ijms-25-13009-s001.zip › ijms-3325799-supplementary.pdf]

Supplementary Material

Monocyte/Macrophage-Specific Loss of ARNTL Suppresses Chronic Kidney Disease-Associated Cardiac Impairment

Yuya Yoshida[1*], Naoki Nishikawa[1], Kohei Fukuoka[1], Akito Tsuruta[2], Kaita Otsuki[1], Taiki Fukuda[1], Yuma Terada[1], Tomohito Tanihara[1], Taisei Kumamoto[1], Ryotaro Tsukamoto[1], Takumi Nishi[1], Kosuke Oyama[3], Kengo Hamamura[1], Kouta Mayanagi[4], Satoru Koyanagi[2], Shigehiro Ohdo[1], Naoya Matsunaga[1*]

[1] Department of Clinical Pharmacokinetics, Faculty of Pharmaceutical Sciences, Kyushu University, 3-1-1 Maidashi Higashi-ku, Fukuoka 812-8582, Japan
[2] Department of Pharmaceutics, Faculty of Pharmaceutical Sciences, Kyushu University, 3-1-1 Maidashi Higashi-ku, Fukuoka 812-8582, Japan
[3] Department of Biological Science and Technology, Faculty of Advanced Engineering, Tokyo University of Science, 1-3 Kagurazaka, Shinjuku-ku, Tokyo 162-8601, Japan
[4] Department of Drug Discovery Structural Biology, Faculty of Pharmaceutical Sciences, Kyushu University, 3-1-1 Maidashi Higashi-ku, Fukuoka 812-8582, Japan
* Correspondence: yoshida@phar.kyushu-u.ac.jp (Y.Y.); matunaga@phar.kyushu-u.ac.jp (N.M.)

Table S1. Primer sets for the construction of *Gpr68* or *Arntl* luciferase reporter plasmids.

	Primers
Gpr68(-1734)-Luc	
Forward	5'-CCCCTCGAGATGGGGTGAAATCTACA-3'
Reverse	5'-ACAAGATCTGTCCACTCTACCCGCGA-3'
Gpr68(-1512)-Luc	
Forward	5'-CCCCTCGAGACAAGACATACCACTGT-3'
Reverse	5'-ACAAGATCTGTCCACTCTACCCGCGA-3'
Gpr68(-1261)-Luc	
Forward	5'-GCCCTCGAGCCTGAGACAATGTCCTG-3'
Reverse	5'-ACAAGATCTGTCCACTCTACCCGCGA-3'
Gpr68(-27)-Luc	
Forward	5'-CCCCTCGAGATCCGTCTCTAGCTACAT-3'
Reverse	5'-ACAAGATCTGTCCACTCTACCCGCGA-3'
Arntl (-1500)-Luc	
Forward	5'- CGGCTCGAGGTGTGCCTTGCAAAGGGC -3'
Reverse	5'- CCCAGATCTCGCAGCCATGCCGACA -3'

Table S2. Primer sets for RT-PCR and ChIP analysis.

Gene		Primers
**Mouse**	***Gpr68***	
	Forward	5'-CGTGGTCATCTTCCTGGCTT-3'
	Reverse	5'-TGGTGAGGAGGAGGGAGAAG-3'
**Mouse**	***aSMA***	
	Forward	5'-GGCACCACTGAACCCTAAGG-3'
	Reverse	5'-ACAATACCAGTTGTACGTCCAGA-3'
**Mouse**	***Col1a2***	
	Forward	5'-AAGGGTGCTACTGGACTCCC-3'
	Reverse	5'-TTGTTACCGGATTCTCCTTTGG-3'
**Mouse**	***Tnfa***	
	Forward	5'-CTGAACTTGGGGGTGATCGG-3'
	Reverse	5'-GGCTTGTCACTCGAATTTTGAG-3'
**Mouse**	***Il6***	
	Forward	5'-CTGCAAGAGACTTCCATCCAG-3'
	Reverse	5'-AGTGGTATAGACAGGTCTGTTGG-3'
**Mouse**	***Arntl***	
	Forward	5'- GGACTTCGCCTCTACCTGTTCA -3'
	Reverse	5'- AACCATGTGCGAGTGCAGGCGC -3'
**Mouse**	***Pu1***	
	Forward	5'- ATGTTACAGGCGTGCAAAATGG -3'
	Reverse	5'- TGATCGCTATGGCTTTCTCCA -3'
**Mouse**	***Col1a1***	
	Forward	5'- TAAGGGTCCCCAATGGTGAGA -3'
	Reverse	5'- GGGTCCCTCGACTCCTACAT -3'
**Mouse**	***Timp1***	
	Forward	5'- GCAACTCGGACCTGGTCATAA -3'
	Reverse	5'- CGGCCCGTGATGAGAAACT -3'
**Mouse**	***Mmp1a***	
	Forward	5'- CCTTGATGAGACGTGGACCAA -3'
	Reverse	5'- ATGTGGTGTTGTTGCACCTGT -3'
**Mouse**	***Tgfb***	
	Forward	5'- TGACGTCACTGGAGTTGTACGG -3'
	Reverse	5'- GGTTCATGTCATGGATGGTGC -3'

**Mouse**	***Vcam1***	
	Forward	5'- AGTTGGGGATTCGGTTGTTCT -3'
	Reverse	5'- CCCCTCATTCCTTACCACCC -3'
**Mouse**	***Sele***	
	Forward	5'- ATGCCTCGCGCTTTCTCTC -3'
	Reverse	5'- GTAGTCCCGCTGACAGTATGC -3'
**Mouse**	***Gpr68* gene (E-box) for ChIP analysis**	
	Forward	5'- AGTAATAAAACTTCATTGCAGTCCC -3'
	Reverse	5'- GTCACTCACCCAATCAATGAAACC -3'
**Mouse**	***Timd4***	
	Forward	5'- GTGTACTGCTGCCGTATAGAGG -3'
	Reverse	5'- TGGTGGTTGGGAGAACAGATG -3'
**Mouse**	***Folr2***	
	Forward	5'- CCTGTCGTACCTCCTTTACCT -3'
	Reverse	5'- TTTGGGCACTTGTTAATGCCT -3'
**Mouse**	***Lyve1***	
	Forward	5'- CTCGTGCAAGACCTTTCCATT -3'
	Reverse	5'- GCCTCGTTGGCTTCTGTGAA -3'
**Mouse**	***Il1b***	
	Forward	5'- TGGATGCTCTCATCAGGACAG -3'
	Reverse	5'- GAAATGCCACCTTTTGACAGTG -3'
**Human**	***GPR68***	
	Forward	5'- TGTACCATCGACCATACCATCC -3'
	Reverse	5'- GGTAGCCGAAGTAGAGGGACA -3'
**Human**	***ARNTL***	
	Forward	5'- AAGGGAAGCTCACAGTCAGAT -3'
	Reverse	5'- GGACATTGCGTTGCATGTTGG -3'
**Human**	***PER1***	
	Forward	5'- GCCAACCAGGAATACTACCAGC -3'
	Reverse	5'- GTGTGTACTCAGACGTGATGTG -3'

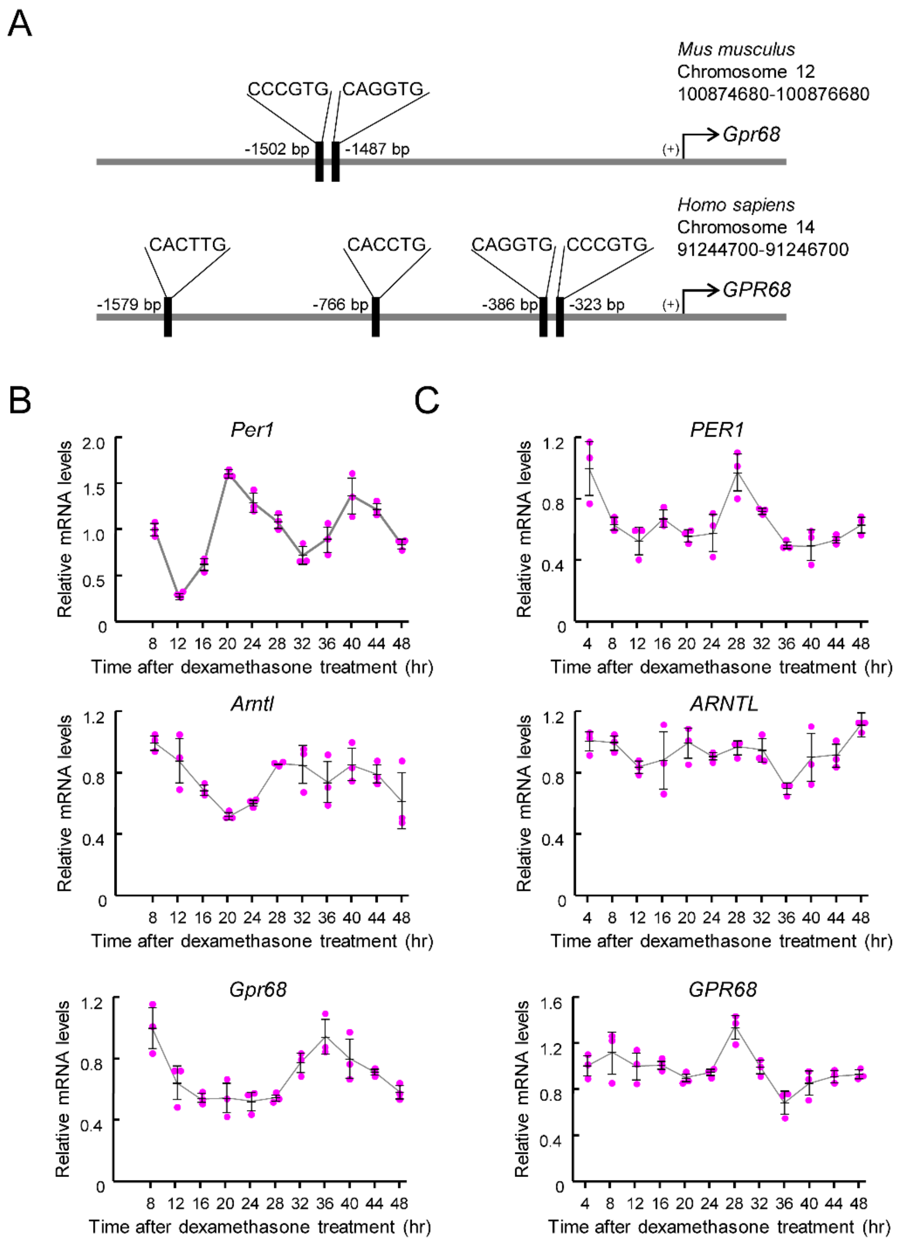

Figure S1. Circadian periodicity of GPR68 expression in human and mouse cells. (A) Schematic representation of the E-boxes within the upstream region of the *GPR68* gene in humans and mice. The numbers indicate the distance from the transcription start site (+1). Black rectangles, E-box. (B, C) Temporal expression profiles of *PER1*, *ARNTL*, *GPR68*, *Per1*, *Arntl*, and *Gpr68* mRNA in RAW264.7 and THP-1 cells after synchronization of the circadian clock. Cells were treated with 100 nM dexamethasone for 2 h to synchronize the circadian clock, and then mRNA levels of *PER1*, *ARNTL*, *GPR68*, *Per1*, *Arntl*, and *Gpr68* were assessed at the indicated time points. There was a significant time-dependent variation in the mRNA levels of *PER1*, *ARNTL*, *GPR68*, *Per1*, *Arntl*, and *Gpr68* ($P < 0.05$; one-way ANOVA). Values are expressed as the mean with SD ($n = 3$).

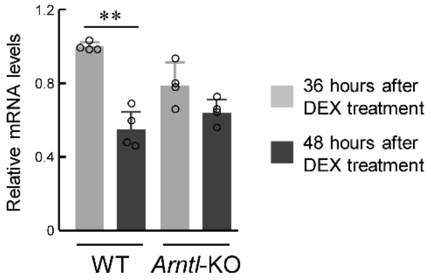

Figure S2. Relationship between GPR68 and ARNTL expression. Expression of *Gpr68* mRNA in *Arntl*-KO RAW264.7 cells after synchronization of the circadian clock. Cells were treated with 100 nM dexamethasone for 2 h to synchronize the circadian clock, and then mRNA levels of *Gpr68* were assessed at 36 and 48 h after dexamethasone treatment. Values are expressed as the mean with SD (n = 4). **, $P < 0.01$ indicates significant differences between the two groups (two-way ANOVA with Tukey–Kramer post-hoc tests).

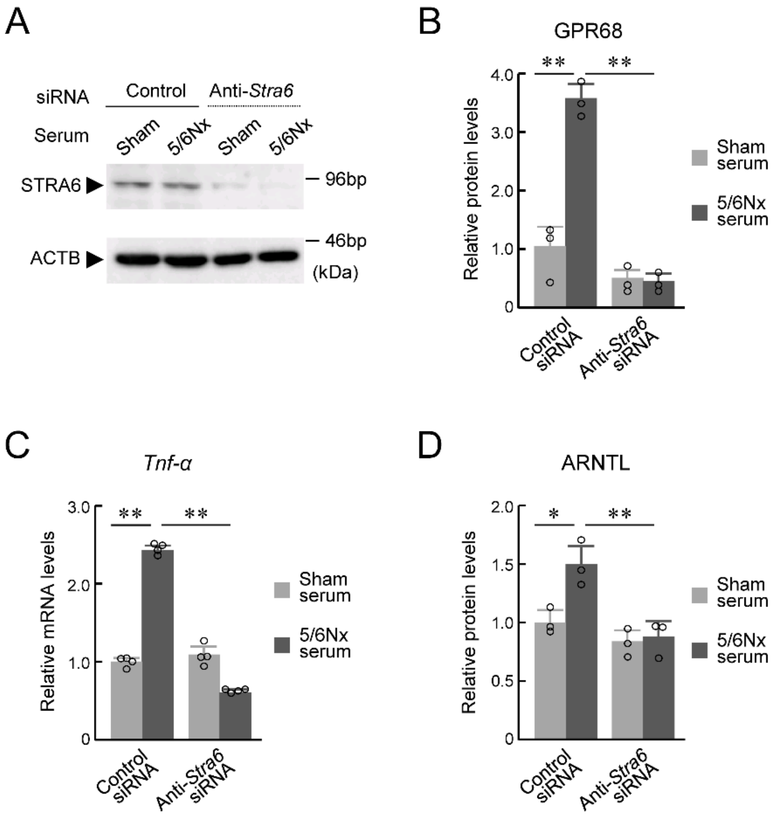

Figure S3. Effect of STRA6 on GPR68 upregulation in RAW264.7. cells treated with serum from 5/6Nx mice. (A) STRA6-downregulated RAW264.7 cells were created by introducing anti-*Stra6* siRNA. STRA6 expression was measured using Western blotting. (B, C, D) Protein levels of GPR68 (B), mRNA levels of *Tnfa* (C), and protein levels of ARNTL (D) in RAW264.7 cells transfected with control or *Stra6*-siRNA were measured after incubation with 10% serum from Sham and 5/6Nx mice for 24 h. Values are expressed as the mean ± SD (n = 3–4). **, $P < 0.01$, *, $P < 0.05$ indicate significant differences between the two groups (two-way ANOVA with Tukey–Kramer post-hoc tests or Student's t-test).

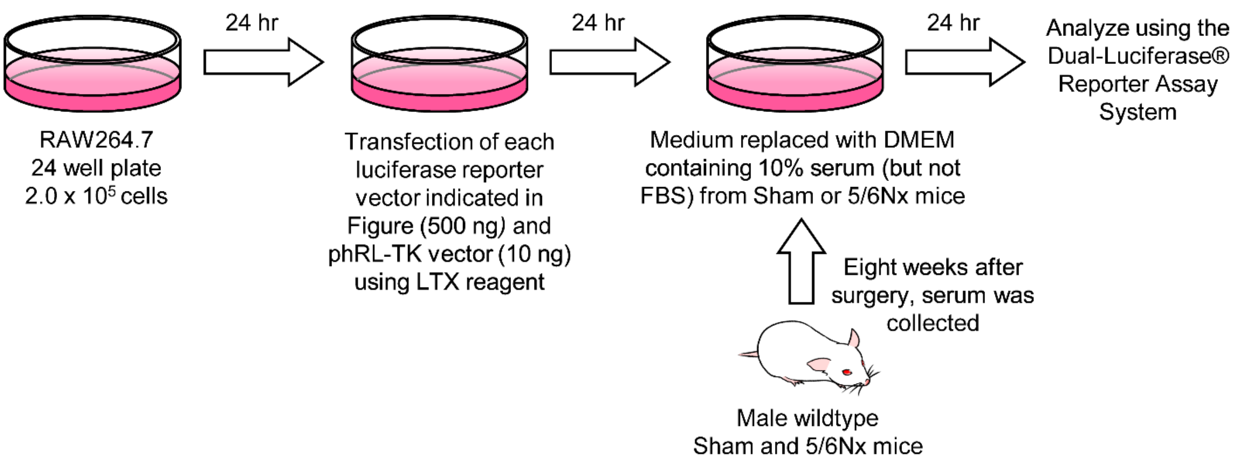

Figure S4. Schematic diagram of the experiment using cultured cells described in Figure 1B and Figure S5.

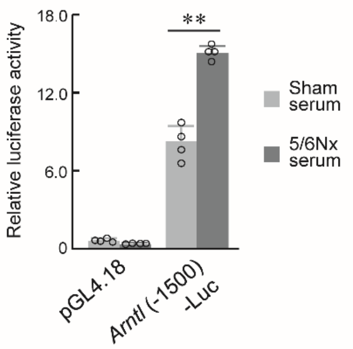

Figure S5. 5/6Nx mouse-derived serum increased the transcriptional activity upstream of *Arntl* in RAW264.7 cells. RAW264.7 cells were transfected with *Arntl* (-1500)-Luc or pGL4.18 and incubated with 10% serum from Sham or 5/6Nx mice for 24 h. Values are expressed as the mean ± SD (n = 4). **, $P < 0.01$ indicates significant differences between the two groups (Student's t-test).

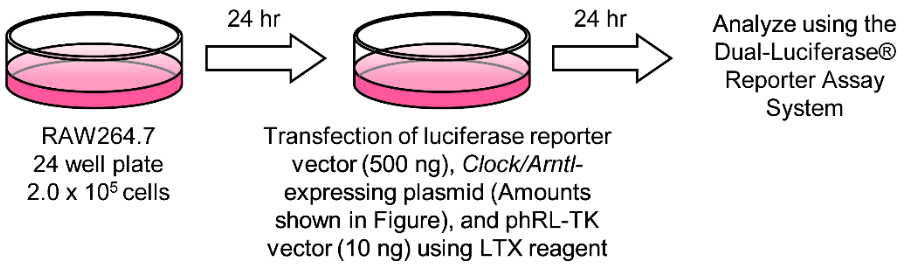

Figure S6. Schematic diagram of the experiment using cultured cells described in Figure 1C.

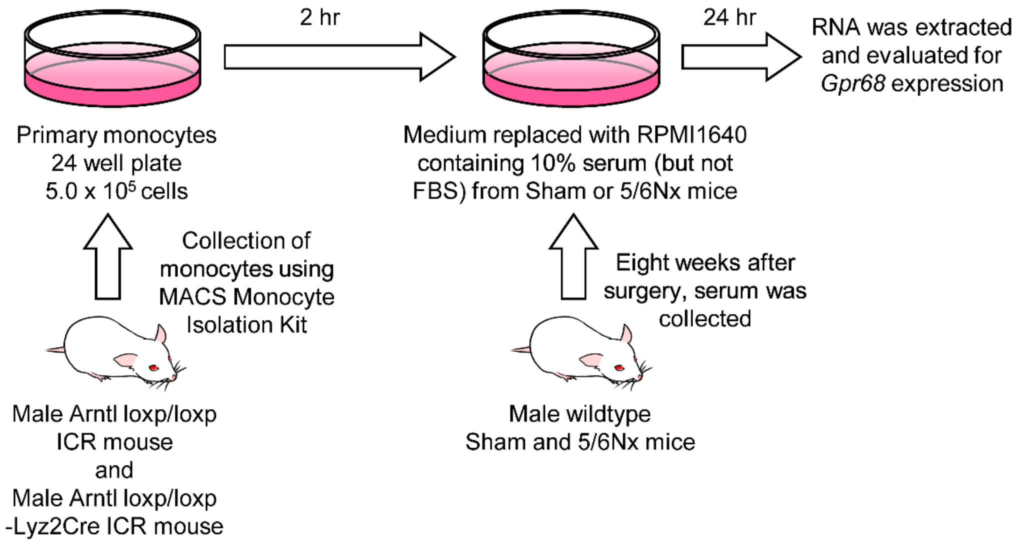

Figure S7. Schematic diagram of the experiment using primary monocytes presented in Figure 1F and G.

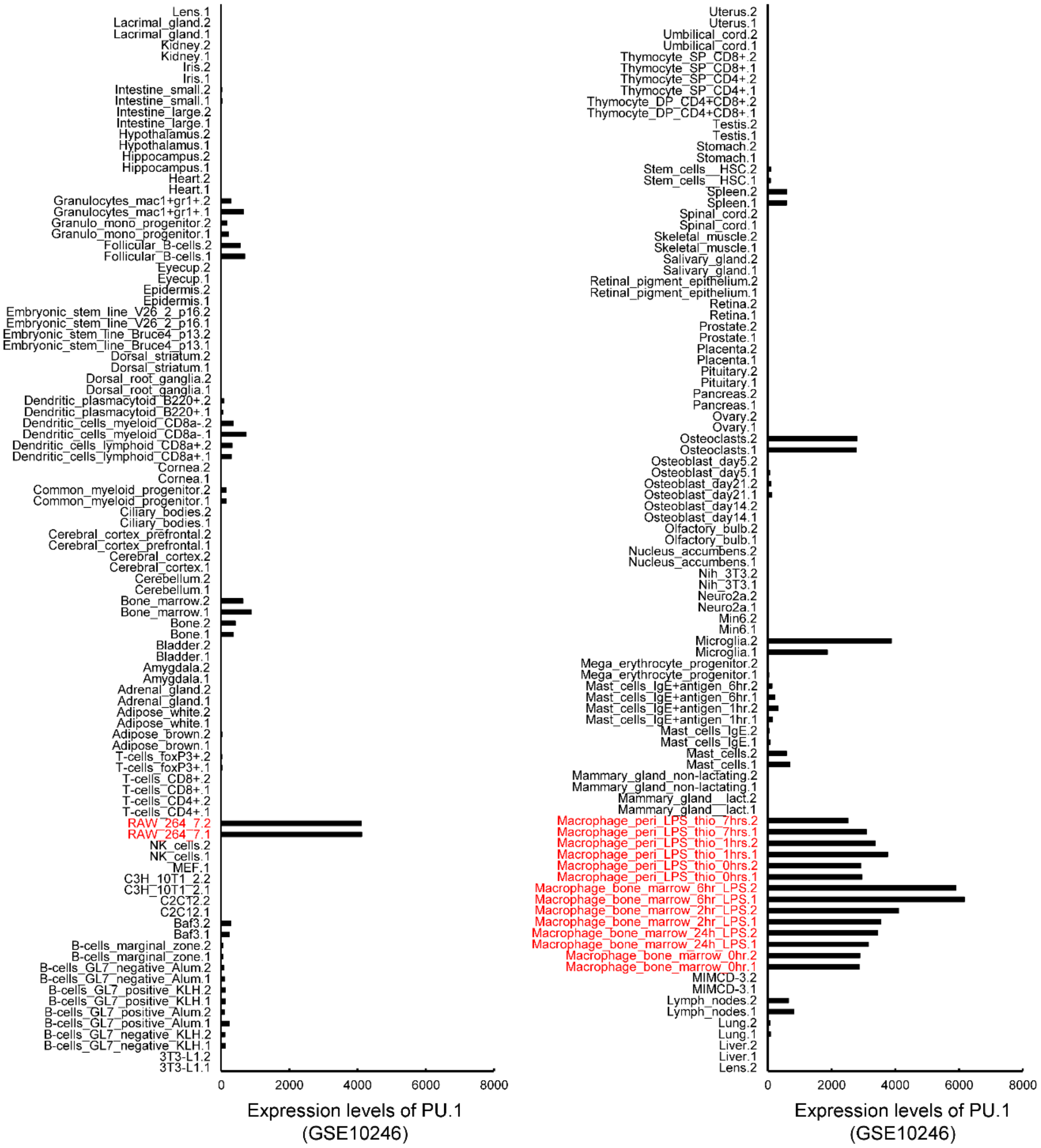

Figure S8. Comparison of PU.1 expression in various organs and cell types. The analyses were performed using the transcriptome database (GSE10246).

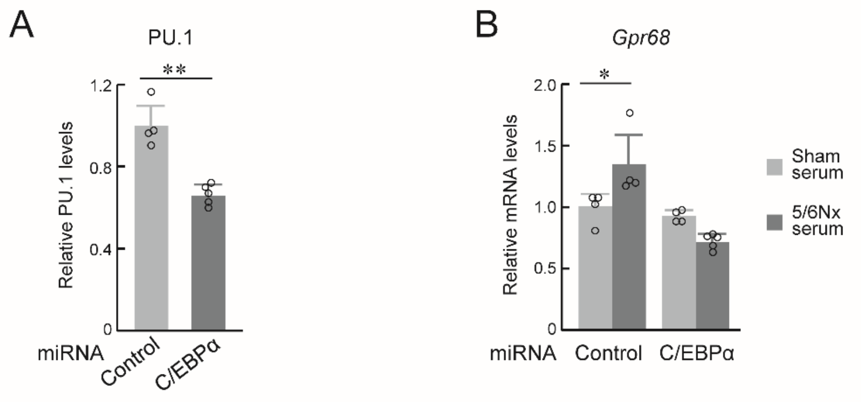

Figure S9. Relationship between PU.1 and GPR68 expression in macrophages. (A) Transfection of mi*Cebpa* to RAW264.7 cells downregulated PU.1 expression. (B) The mRNA levels of *Gpr68* in RAW264.7 cells transfected with control miRNA or mi*Cebpa*-expressing vectors were measured after incubation with 10% serum from Sham or 5/6Nx mice for 24 h. Values are expressed as the mean with SD (n = 4–5). **, $P < 0.01$ indicates significant differences between the two groups (two-way ANOVA with Tukey–Kramer post-hoc tests or Student's t-test).

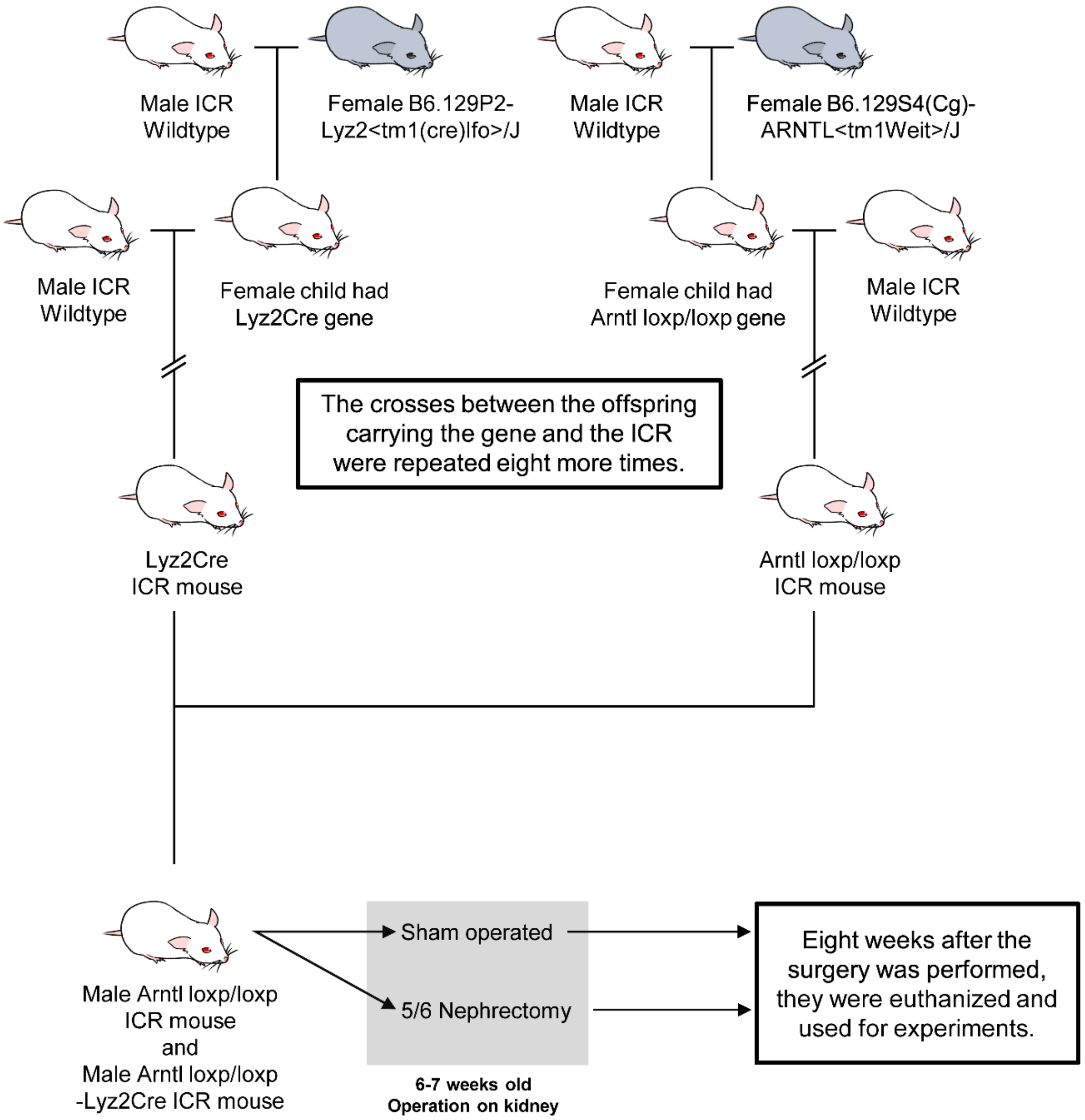

Figure S10. Schematic diagram of experimental animal preparation. Lyz2Cre ICR mice, which had Cre knock-in at the Lysozyme 2 locus, and *Arntl* loxp/loxp ICR mice, which had loxp sequences inserted at the *Arntl* locus, were generated by crossing B6.129P2-Lyz2<tm1(cre)Ifo>/J and B6.129S4(Cg)-ARNTL<tm1Weit>/J with Jcl:ICR mice for more than nine generations. Among the assortments obtained by crossing the Lyz2Cre ICR mice with ARNTL loxp/loxp ICR mice for three generations, male mice carrying only *Arntl* loxp/loxp were designated as monocyte ARNTL-expressing (ARNTL +/+) mice, and male mice carrying *Arntl* loxp/loxp and Lyz2Cre were used for experiments as monocyte-specific ARNTL-deficient (ARNTL -/-) mice. CKD mouse models were generated using a two-step 5/6Nx model at five or six weeks of age. During the first surgery, two-thirds of the left kidney was removed by cutting off both poles. Then, seven days later, the right kidney was excised. The mice were housed for eight weeks after the operation until CKD developed. Sham mice underwent laparotomies on the same day the experimental mice underwent 5/6Nx surgery.

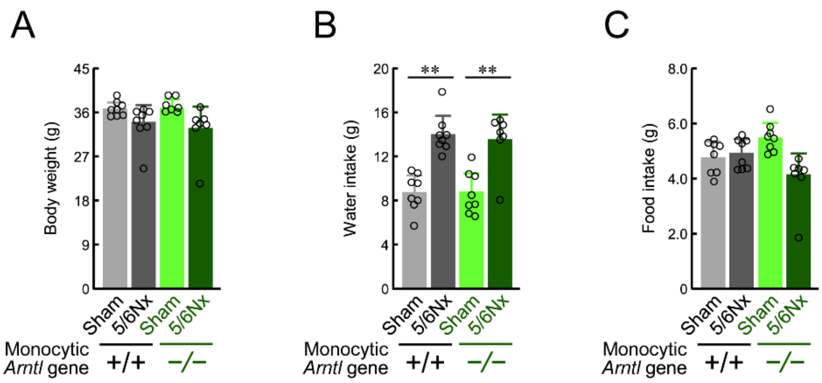

Figure S11. Changes in the body weight (A) and amount of water (B) or food (C) intake of *ARNTL* +/+ or *ARNTL* -/- Sham and 5/6Nx mice eight weeks after the 5/6Nx operation. Values are expressed as the mean with SD (n = 6–8). **, $P < 0.01$ indicates significant differences between the two groups (two-way ANOVA with Tukey–Kramer post-hoc tests).

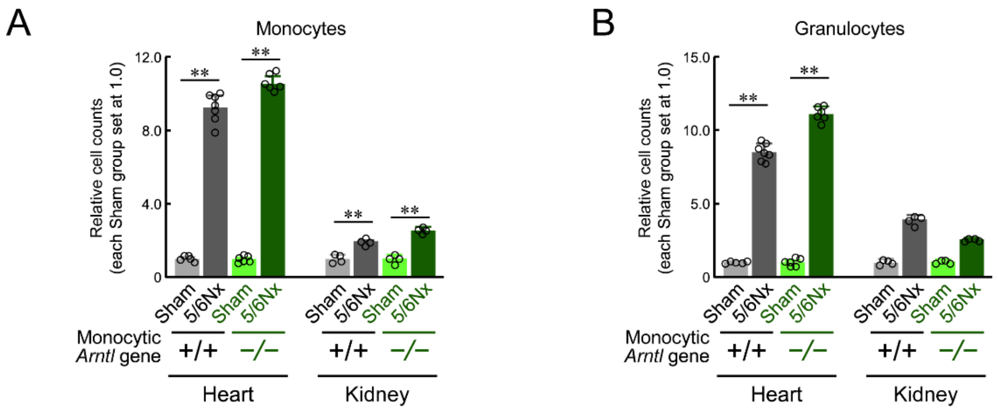

Figure S12. The number of monocytes (A), and granulocytes (B) in each organ. Values are expressed as the mean with SD (n = 4–6). The mean value of the sham-operated *ARNTL* +/+ group in each organ was set as 1.0. **, $P < 0.01$ indicates significant differences between the two groups (two-way ANOVA with Tukey–Kramer post-hoc tests).

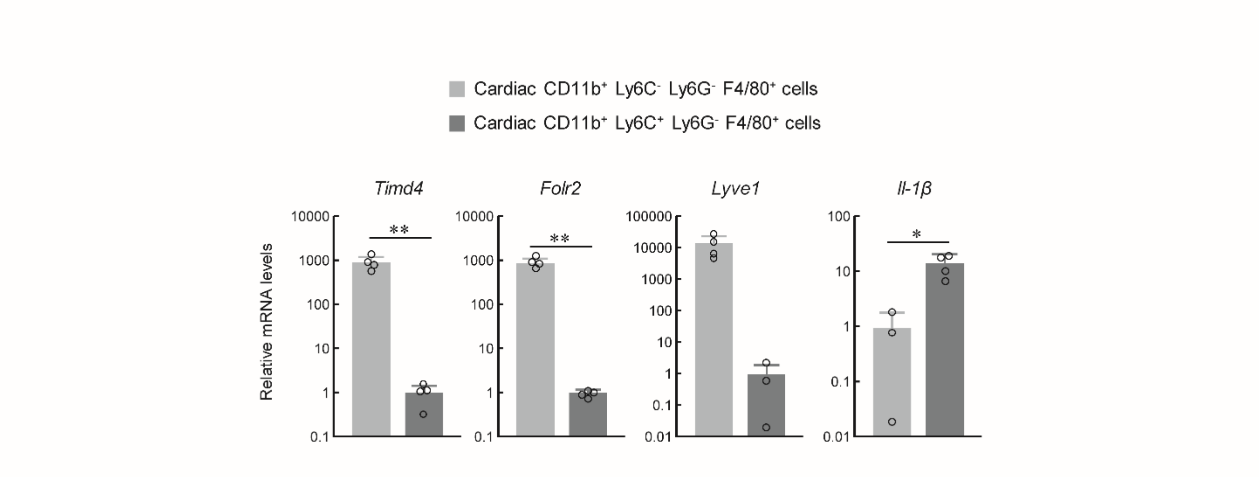

Figure S13. mRNA levels of *Timd4*, *Folr2*, *Lyve1*, and *Il1b* in the cardiac F4/80$^+$/Ly6G$^-$/CD11b$^+$/Ly6C$^+$ or F4/80$^+$/Ly6G$^-$/CD11b$^+$/Ly6C$^-$ cells of *ARNTL* +/+ 5/6Nx mice. The mean value of the F4/80$^+$/Ly6G$^-$/CD11b$^+$/Ly6C$^+$ group (*Timd4, Folr2,* and *Lyve1*) or F4/80$^+$/Ly6G$^-$/CD11b$^+$/Ly6C$^-$ group (*Il1b*) was set to 1.0. Values are expressed as the mean with SD (n = 4). **, $P < 0.01$ indicates significant differences between the two groups (Student's t-test).

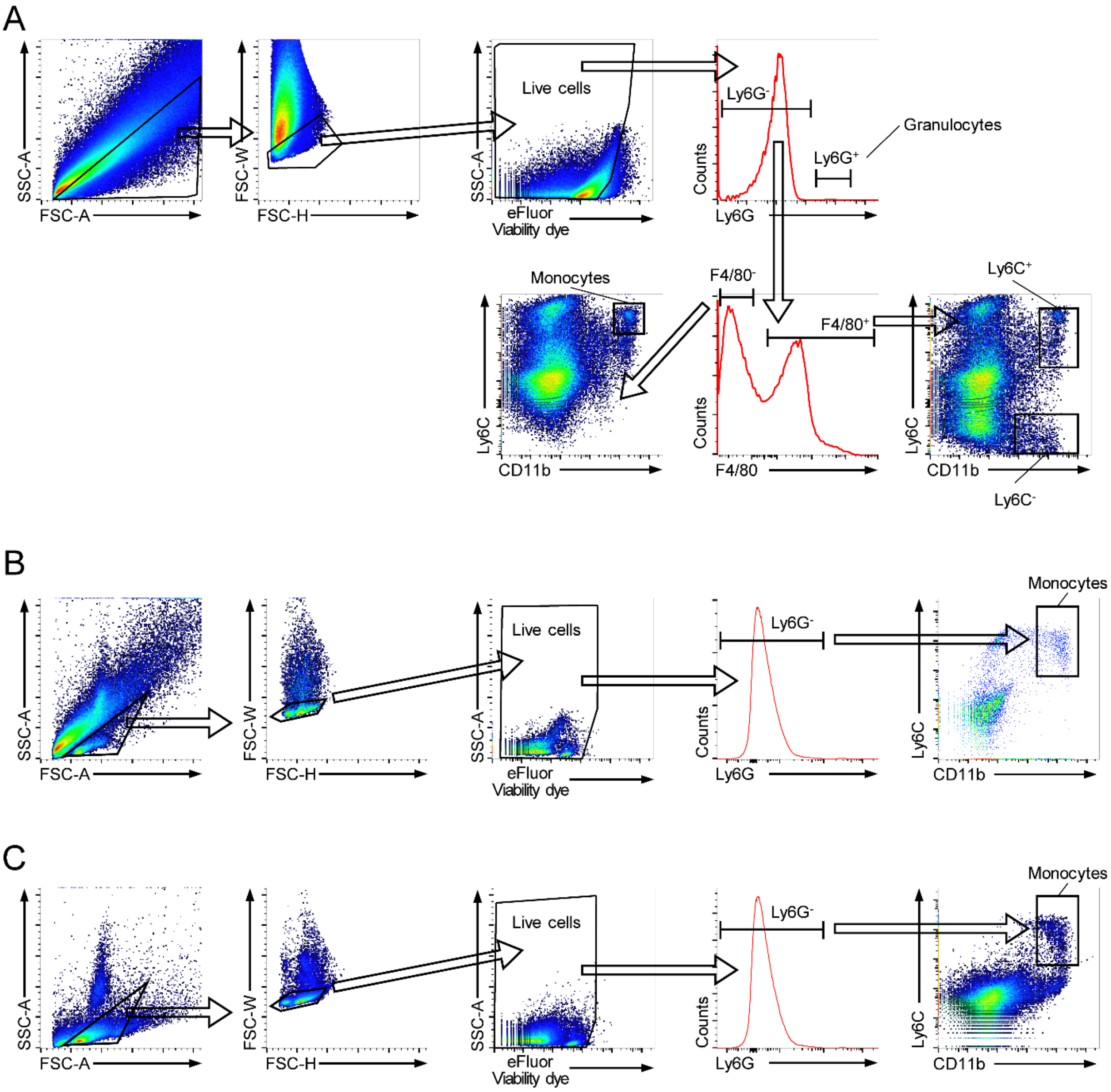

Figure S14. Gating strategies used for flow cytometry analysis. (A) Gating strategy for sorting cardiac cells. (B) Gating strategy for sorting Ly6G$^-$/CD11b$^+$/Ly6C$^+$ cells (monocytes) from the circulating blood of mice. (C) Gating strategy for sorting Ly6G$^-$/CD11b$^+$/Ly6C$^+$ cells (monocytes) from the spleens of mice.
